# Isolation and Molecular Characterisation of *TtDro1A* and *TtDro1B* Genes from *Triticum turgidum* Subspecies *durum* and *turgidum*, Study of Their Influences on Seedling Root Angles

**DOI:** 10.3390/plants11060821

**Published:** 2022-03-19

**Authors:** Yolanda Loarce, Alejandra Cabeza, Rodrigo Cañas, Juan M. González

**Affiliations:** Departamento de Biomedicina y Biotecnología, Universidad de Alcalá, 28805 Alcalá de Henares, Spain; yolanda.loarce@uah.es (Y.L.); acabeza@eead.csic.es (A.C.); rodricober@gmail.com (R.C.)

**Keywords:** *Dro1* gene, root angle, root system architecture, RSA

## Abstract

Durum wheat (*Triticum turgidum,* 2n = 4x = AABB) includes several subspecies with differential characteristics in their root system architecture (RSA). Subspecies *durum* has longer and more vertical roots, while subspecies *turgidum* has smaller and shallower roots. The homeologous genes *TtDro1A* and *TtDro1B* of both subspecies have been identified and found to differ in their sizes, sequences and the proteins they encode. To determine whether there is a relationship between the level of expression of these two genes and the angle adopted by the roots of durum wheat seedlings, their expressions has been studied by RT-qPCR, both in the primary seminal root and in the other seminal roots. The results of the analyses showed that the *TtDro1A* gene is expressed 1.4 times more in the primary seminal root than in the other seminal roots. Furthermore, this gene is expressed 2.49 to 8.76 times more than *TtDro1B* depending on root type (primary or seminal) and subspecies. There are positive correlations between the expression ratio of both genes (*TtDro1A*/*TtDro1B*) and the mean of all root angles, the most vertical root angle and the most horizontal root angle of the seedlings. The higher the expression of *TtDro1B* gene, the lower the root growth angles.

## 1. Introduction

Durum wheat (*Triticum turgidum* Desf. 2n = 4x = 28, AABB genomes) includes several subspecies such as *durum* and *turgidum* [1,2,3]. This species is of great economic importance since is used to produce important food products such as pasta and couscous. The main durum-growing regions are the Middle East, Southern Europe, North Africa, the former Soviet Union, North America, and India. Worldwide, durum is grown on approximately 17 million hectares and the production in 2019 was about 38.1 million tons [4,5]. Although the majority of durum wheat cultivation is rain-fed, the production and yield of currently cultivated varieties may decrease considerably due to water shortages because of the climate change [6,7,8]. It is necessary to obtain new varieties capable of developing and maintaining the yield with a lower contribution of rainwater and, in this scenario, the root system of the plants has a great importance since the root is the organ that the plants use to capture mineral nutrients and water [9,10,11,12].

The number, length, and angle of roots at which they grow into the soil has been called root system architecture (RSA) and will largely determine the plant ability to reach nutrients and water [13,14]. Several authors have demonstrated the importance of the type of root system in the intake of nitrogen, phosphorus, or water on crop yield [15,16,17,18], and how deeper root systems increase production under drought conditions [10,19,20,21].

It has been proposed that through genetic breeding programs, RSA can be remodelled so that the roots are able to reach deeper, wetter areas of the soil, making survival and plant growth possible [22,23,24,25,26]. However, RSA is a complex topic and governing genes, as well as the environmental factors, influence the development of the root system. Several studies have attempted to elucidate the genetic factors underlying the establishment of RSA in cereals showing the complex genetic control associated with RSA, mostly regulated by a set of genes with small effect, although QTLs that individually explain up to 30% of the phenotypic variation in rice and maize and up to 50% in wheat, have also been described [23,27,28,29,30,31,32,33,34].

One of the most important features of RSA is the angle at which the roots grow, which will determine whether the roots will be shallow or deep and therefore have better access to nutrients that remain at the soil surface such as phosphorus, or water resources and nitrogen that accumulates in deeper layers [35,36]. Thus, a narrow root angle in rice was shown to improve yield by up to 10% when water was not limiting [35].

In cereals, the seminal roots are used by the plants both in the first weeks of growth and in advanced stages of development, being the primary seminal root that go deeper into the soil [37,38]. The ability of the seedling to develop its roots and reach the deeper soil layers where water is more abundant is a critical characteristic for plant establishment and subsequent vegetative development [13,39,40,41]. Sanguineti et al. [42] found that the length and weight of the seminal roots at an early growth stage provide useful clues about early vigour, a desirable trait for the improvement of water use efficiency and yield in wheat. A previous work has shown that the angle of the seminal roots in the seedling stage is correlated with the RSA in the mature plant [15], and Robinson et al. [43] found a correlation ranging from −0.21 to 0.36, between root angle and yield traits in a panel of 216 two-rowed spring barley genotypes.

Several QTLs and genes associated with rice roots had been described [20,36,44,45]. These authors identified the *Dro1* (Deeper Rooting 1) locus from the DNA of two rice cultivars showing polymorphism for root inclination [20]. The study was able to associate the polymorphism with a mutation responsible to produce a truncated Dro1 protein in the cultivar with smaller and shallower roots, which was not present in the cultivar with longer and deeper roots. Overexpression of *Dro1* in rice or its introduction by backcrossing into shallow-rooted cultivars caused changes in the RSA, decreasing the root growth angle and thus producing deeper rooting. The influence of *Dro1* has also been shown in other plants such as *Arabidopsis*, where mutation of *AtDro1* produced more horizontal roots, or in *Prunus domestica* where overexpression of *PpeDro1* produced phenotypes with deeper roots [46]. It is expected that, as in rice, the homologous loci to the *Dro1* gene play a role in determining the root angles in durum wheat. Furthermore, as it is a polyploid plant, the genes will be duplicated, and their involvement in the root angles may not necessarily be the same, which will require the study of the loci individually.

Ruiz et al. [47] conducted a study of the RSA in the Spanish durum wheat core collection found that the seedlings showed a large variability with respect to the mean of all root angles, the most vertical root angle and the most horizontal root angle. In addition, they found that the subspecies *durum* has larger and more vertical roots than the subspecies *turgidum*. In the present work we aim to investigate whether the difference in the RSA between the two subspecies is determined, at least in part, by the durum wheat genes homeologous to the rice *Dro1* gene. For this purpose, two genotypes with very different RSAs were selected, one belonging to the subspecies *durum* and the other to *turgidum*, with the following objectives: (1) to identify and characterise the genes homeologous to the rice *Dro1* gene, (2) to study by RT-qPCR the expression of these genes in the seminal roots of seedlings of a collection of genotypes of *durum* and *turgidum* subspecies, and (3) to determine the possible relationship between their expression level and root angles in seedlings of *durum* and *turgidum* subspecies.

## 2. Results

### 2.1. RSA Analysis

**Figure 1:**
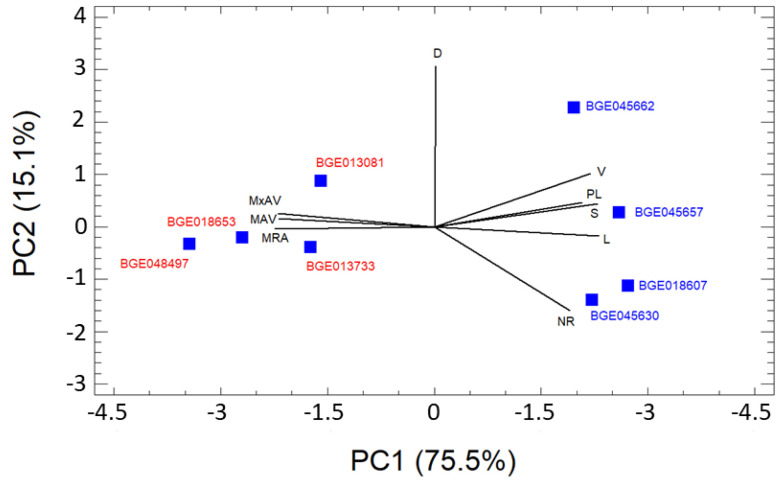
Biplot of the PCA two first principal components. Trait vectors of the RSA and the location of genotypes of the subsp. *durum* (blue) and turgidum (red) are showed. (NR) number of roots, (TRL) total root, (PRL) primary seminal length, (S) total root area, (V) total root volume, (DS) mean root diameter, (MRA) mean of root angles respect to the vertical, (MAV) minimum root angle and (MxRA) maximal root angle.

Mean values of the nine RSA variables in each of the eight genotypes of *T. turgidum* (Table 1) were used to perform a Principal Component Analysis (PCA). In the Figure 1, a biplot of the first two main components is shown. The first principal component explains 75.5% of the variance and separates the genotypes as a function of variables related to root angles on the one hand, and length, volume, and surface of the roots on the other.

**Figure 2:**
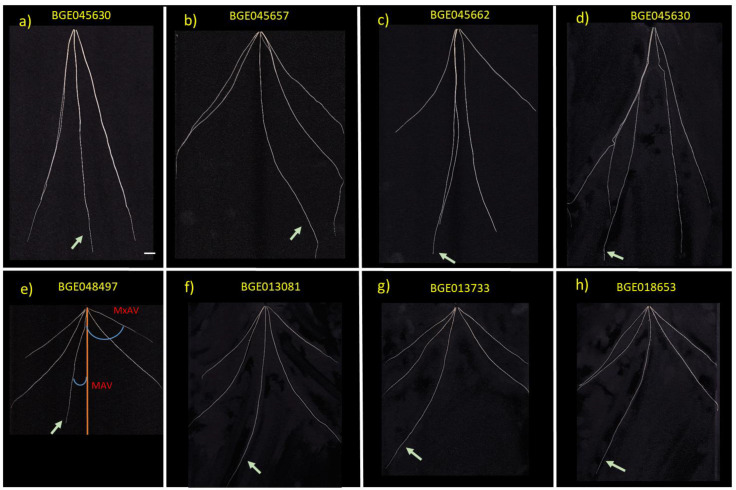
Examples of root development of the four genotypes of *T. turgidum durum*: (**a**–**d**); and the four of *T. turgidum turgidum*: (**e**–**h**). (**e**) shows how the maximum (MxAV) and minimum angles (MAV) of the roots with respect to the vertical were measured. The arrows point to the seminal primary roots.

There is a clear differentiation between genotypes according to the subspecies they belong to. Thus, subspecies *durum* has a larger root system with more vertical roots while the roots of subspecies *turgidum* have a shorter overall length and are shallower. Figure 2 shows some examples of the RSA of the eight genotypes studied.

**Figure 3:**
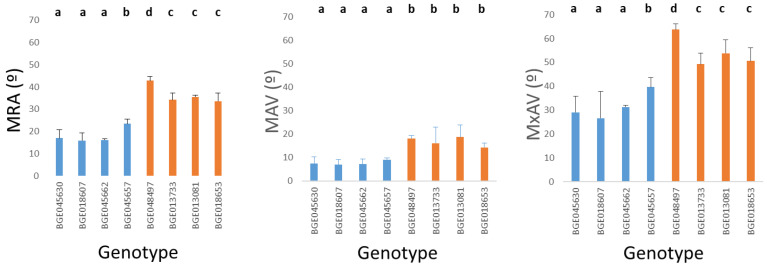
Mean values of the MRA (mean of root angles), MAV (minimum root angle), MxAV (maximal root angle). Results of the Fisher’s LSD test of the three variables related with the root angle. Genotypes with the same letter have no statistically significant differences at *p* < 0.05. Blue and orange bars correspond to *durum* and *turgidum* subspecies, respectively. The error bars show SD (standard deviation).

Analysis of variance (ANOVA) taken the genotype as independent factor were performed for each of the three variables related with the root angles. The results were highly significant (*p* < 0.05). Figure 3 is a graphical representation of MRA, MAV and MxAV in the eight genotypes, including the results of Least Significant Difference test (LSD).

### 2.2. Identification, Cloning and Analysis of the TtDro1A and TtDro 1B Genes of T. turgidum

For the identification, cloning and analysis of the *TtDro1* genes of *T. turgidum*, the genotypes BGE045630 from subspecies *durum* and BGE048497 from subspecies *turgidum* were selected on the basis that they have long deep roots and short shallow roots, respectively.

**Figure 4:**
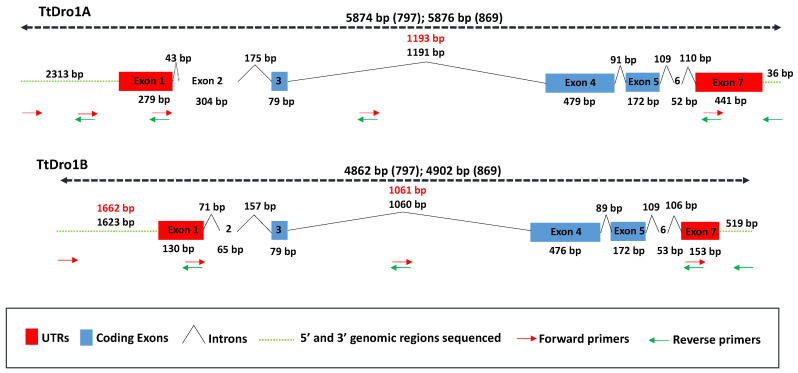
Diagram of the subgenomic regions containing the sequenced *durum* wheat *TtDro1* genes from BGE045630 (abbreviated as 797) y BGE048497 (abbreviated as 869) genotypes. UTR regions, introns and exons are identified. The regions of the primers used are indicated with arrows, forward primers in red and reverse primers in green.

Firstly, the rice Dro1 gene sequence [20] was used in a BLAST search with the *Triticum aestivum* sequences included in the Ensembl Plants database [48] (http://plants.ensembl.org/Triticum_aestivum/Tools/Blast. Accessed on 18 March 2021). The *T. aestivum* homologous sequences located in the 5A and 5B chromosomes were used for PCR subgenomes specific primers design. The Figure 4 shows a representation of the amplified gene regions (Promoters, UTRs, exons, and introns) and the position of the primers used in PCR reactions. The amplified regions of both genomes of each of the lines were aligned for comparison (Figure S1). The information for annotation of sequences (UTRs, exons, and introns) were obtained from the *T. aestivum* cDNA clones included in Ensembl Plants database.

The subgenomic A regions containing the *TtDro1A* homologous sequence span 5874 and 5876 bp for the BGE045630 and BGE048497 genotypes, respectively (GenBank accession numbers: MZ151530, MZ151531, MZ151532, MZ151533). Both sequences are identical except for two extra bases in the larger intron in the BGE048497 genotype and therefore have no effect on the amino acid sequence of the protein. The *TtDro1B* homologous genomic regions sequenced from B genome were shorter, spanning 4862 bp for BGE045630 and 4902 for BGE048497. In addition to one more bp in the longer intron in the BGE048497 genotype, the alignment showed two different bp in exon 4 that involves the change of two amino acids in the proteins. More differences were found between the 5′ regions of *TtDro1B* coding sequences in both genotypes, being striking the presence of an insertion of 38 bp in the BGE048497 genotype. This insertion was catalogued by the CENSOR software [49] as a partial MITE (Miniature Inverted-repeat Transposable Element) element belonging to the Tc1-Mariner class, specifically to the Stowaway superfamily and Hades family.

### 2.3. Analysis of TtDro1A and TtDro1B Proteins

**Figure 5:**
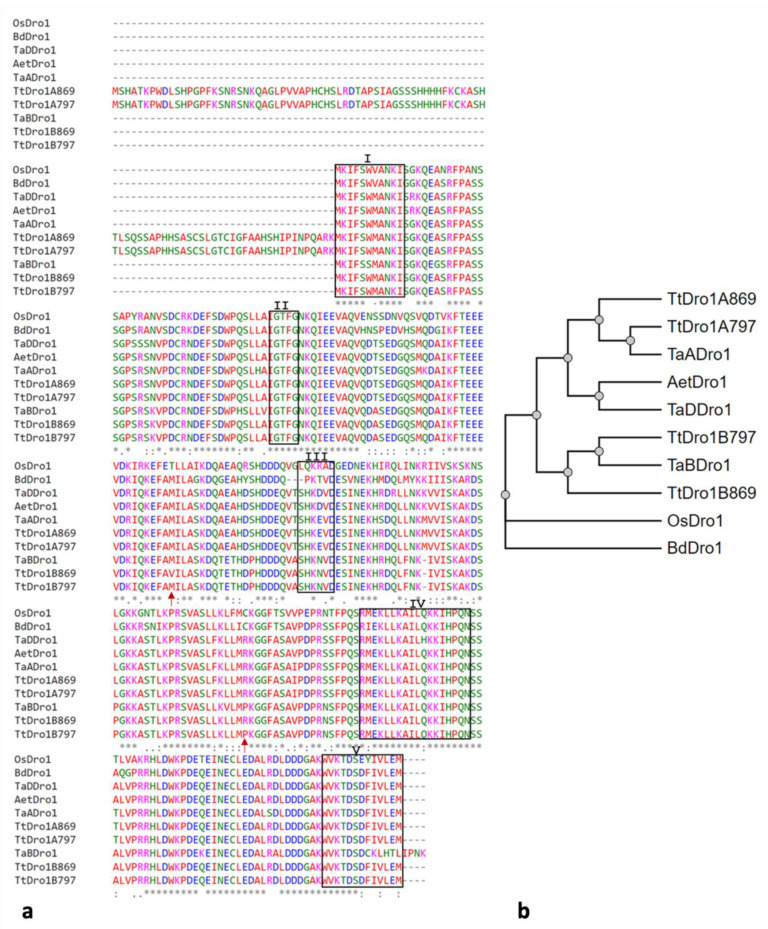
(**a**) Alignment of amino acid sequences of Dro1 cereal proteins. The species and accession numbers used were: rice (OsDro1, Q69P88), *Brachypodium* (Bd Dro1 XM003578131), *T. aestivum* (TaADro1, *MK639011*; TaBDro1, *MK639010*; TaDDro1, *MK639012*), *Aegilops speltoides* (*AetDro1 XM020332849*) and *T. turgidum* (MZ151530, MZ151531, MZ151532, MZ151533). The numbered black boxes show the characteristic domains of the IGT gene family. The arrows indicate the 2 differences between the Dro1B proteins between genotypes BGE045630 and BGE048497, abbreviated as 797 and 869, respectively. (*) Identical amino acids in all analysed proteins. (**b**) Phylogenetic tree from the amino acid sequences of Dro1 proteins.

The gene sequences obtained from BGE045630 and BGE048497 genotypes were translated into the amino acids sequence coded by them. An alignment of these sequences (Figure 5a) together with those of Dro proteins from other cereals was used to construct a phylogenetic tree (Figure 5b).

The alignment of the sequences showed that *TtDro1A* and *TtDro1B* code different proteins but with the characteristic motifs (I-V) for the IGT gene family. The most substantial difference is that protein A is longer, with 96 additional amino acids at the N-terminal end that are not present in the protein encoded by the *TtDro1B* gene nor in the other Dro1 proteins included in the alignment. These results would indicate that it is the *TtDro1B* gene that encodes a protein with a function similar to that encoded by the rice *Dro1* gene. In the rest of the amino acid sequence the proteins of both sub-genomes of *durum* wheat showed 8 more differences. However, no differences were found between subgenome A protein. Two missense change were found between *TtDro1B* proteins from both genotypes, valine for methionine and arginine for proline, respectively (Figure 5a). The PROVEAN software tool [50] was used to predict if these two amino acid substitutions had an impact on the biological function of the protein, being the variants catalogued such as neutral. Therefore, the two amino acid changes in the TtDroB proteins do not seem to be responsible for the different angles of inclination shown by the roots of the two genotypes.

### 2.4. TtDro1A and TtDro1B Genes Expression in Roots of T. turgidum Subsp. durum and turgidum

Since the differences between the proteins encoded by the *TtDro1A* and *TtDro1B* genes did not seem to be responsible for the differences in the root angles of the durum and *turgidum* genotypes, we analysed by RT-qPCR assay whether the differential expression of any of the *TtDro1* genes could be related to the different phenotype in both subespecies.

**Figure 6:**
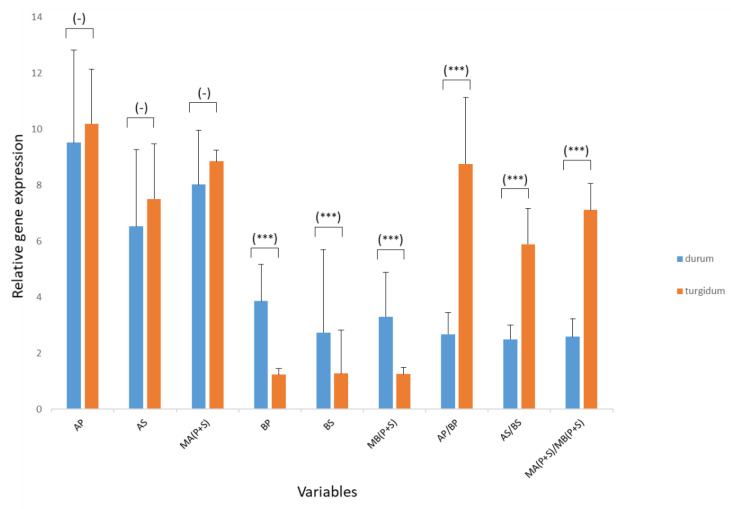
Graphic representation of the nine variables analysed of *TtDro1A* and *TtDro1B* gene expression in both subspecies. Blue column corresponds to durum and orange column to *turgidum*. The error bars show SD (standard deviation). (-) indicates that there are no significant differences between the corresponding means. (***) indicates that there are significant differences between the corresponding means, *p* < 0.005. AP, AS, BP and BS: expression of the *TtDro1A* and *TtDro1B* genes in the primary root (P) and the others seminal (S). MA(P + S): average expression of the *TtDro1A* gene in the primary root and the seminal roots. MB(P + S): average expression of the *TtDro1B* gene in the primary root and the seminal roots. AP/BP: ratio between expressions of the genes *TtDro1A* and *TtDro1B* in the primary root. AS/BS: ratio between expressions of the genes *TtDro1A* and *TtDro1B* in the seminal roots. M(P + S)/MB(P + S): ratio between the average expressions of the genes *TtDro1A* and *TtDro1B* in the primary and the seminal roots.

RT-qPCR of *TtDro1A* and *TtDro1B* was performed in three replicates on both primary and seminal roots of two-day-old seedlings in each of the eight genotypes of subspecies *durum* and *turgidum*. Nine variables were annotated (AP, AS, BP, BS) or calculated (MA(P + S), MB(P + S), AP/BP, AS/BS, MA(P + S)/MB(P + S) as indicated in the Material and Methods section, and their mean values and standard deviation are shown in Table 2. Figure 6 is a representation of these values in both subspecies and the results of the *t*-Student test performed between the corresponding means.

*TtDro1A* is expressed more than *TtDro1B*. The greatest difference in expression between *TtDro1A* and *TtDro1B* was 8.76 (AP/BP) observed in the primary root of subspecies turgidum (Table 2), and the smallest difference in expression (2.49) was observed in the secondary roots of the subspecies *durum*. All variables showed highly significant differences between the two subspecies, except *TtDro1A* gene expression in the primary root, seminal roots and in the mean expression of both root types. [MA(P + S)] (Figure 6).

Considering overall the eight genotypes of the two subspecies of *T. turgidum*, the *TtDro1A* gene is expressed 1.4 times more in the primary root than in the seminal roots (AP/AS = 9.85/7.01) (Table 2). It is also noteworthy that *TtDro1A* and *TtDro1B* are differentially expressed in *T. turgidum*, with *TtDro1A* gene expression being 4.85 times higher than *TtDro1B* (MA(P + S)/MB(P + S) (Table 2). *t*-Student was also applied to compare the expression of *TtDro1A* and *TtDro1B* genes according to the type of root, the ratio (AP/BP)/(AS/BS), and the ratio of average expressions of the genes [MA(P + S)/MB(P + S)], in the primary and the seminal roots, and the results are shown in Table 3.

### 2.5. Study of Correlation between Root Angles and the Expression of the Genes TtDro1A and TtDro1B, in Triticum turgidum

Correlations between variables of the RSA measuring root angles, and expression values of TtDro1A and TtDro1B gene were calculated as follows: the minimum angle (MAV) with respect to TtDro1A and TtDro1B genes expression in the primary root and also with respect to the AP/BP ratio; the maximum angle (MxAV) with respect to TtDro1A and TtDro1B genes expression in the seminal roots and also with respect to the AS/BS ratio; and the mean value of all angles (MRA) with respect to medium expression of the TtDro1A or TtDro1B genes in the primary root and the seminal roots, and with respect to the MA(P + S)/MB(P + S) ratio. Table 4 shows the results obtained. The three positive and highly significant correlations obtained indicate that the greater the expression of the TtDro1B gene with respect to the TtDro1A gene is, the more vertical the roots are.

## 3. Discussion

Varieties of cultivated species with a deep root system have the potential to obtain water resources in underground layers, especially when rainfall is scarce. Landraces and old varieties are the main sources of variability that can be used in breeding programs since they present a great variability of phenotypes of the root system [51,52]. Ruiz et al. [47] studying the Core Collection of Spanish *durum* wheat have demonstrated the existence of a large variability in the RSA and, more specifically, in the root growth angle of the seedlings.

In the present work, the eight genotypes of *T. turgidum* analysed belonging to the subspecies *durum* and *turgidum* have differences in their root system (Table 1). The PCA study (Figure 1) showed that both subspecies have clearly different RSA both in terms of roots size, and root angles to the vertical. Thus, the subspecies *durum* has longer and deeper roots while the subspecies *turgidum* has shorter and shallower roots. Furthermore, the ANOVAs analysis of the three variables related to the angle (MRA, MAV and MxAV) and the corresponding LSD tests confirmed the differential behaviour of both subspecies (Figure 3). These differences may be due, at least in part, to differences in the *TtDro1* gene that controls root inclination as demonstrated by Uga et al. [20] and Kitomi et al. [36] in rice.

From genotypes BGE045630 and BGE048497, belonging to subspecies *durum* and *turgidum*, we have carried out molecular characterisation of the *TtDro1* genes of the A and B subgenomes of *Triticum turgidum*, which are homologous to *Dro1* of rice described by Uga et al. [20]. These genes have shown that the small differences founded in the exonic sequences do not appear to be responsible for the different RGA of both subspecies. The Dro1A-proteins of both lines were identical, and the Dro1B-proteins differed in the substitution of the amino acid proline by leucine (Figure 5) that was classified as neutral by the PROVEAN software tool [50].

The most important difference in the sequence of the *TtDro1B* genes of both lines is the presence of an incomplete MITE element in the 5′UTR region of genotype BGE048497 that is not present in BGE045630, which could influence gene expression. The presence of this mobile element is not surprising because, transposable elements are very abundant in plants, that have evolved different mechanisms to tolerate the presence of Transposable Elements (TE) into or near genes with variable influence on gene [53]. In the case of wheat group species, TE might comprise up to 90% of the genome [54].

BLAST analysis in the Esembl Plants database showed that the proteins encoded by the *TtDro1A* and *TtDro1B* genes were different although both have the typical elements of the IGT family [46]. The *Dro1* genes are orthologous to the LAZY genes that give name to one of the clades of the IGT family and that are involved in shoot and root gravitropism in dicots and monocots plants [55,56]. The LAZY clade differs from the TAC1 clade by the conserved C-terminal domain V containing an EAR motif (Ethylene-responsive element binding factor-associated Amphiphilic Repression). EAR motif-mediated transcriptional repression is the main form of transcriptional repression identified in plants and the proteins containing them can suppress transcription by interacting directly with the promoter as transcription factors, by interacting with other co-repressors such as TOPLESS, or by recruiting histone modifying proteins [57,58]. Surprisingly, TtDro1A and TtDro1B have the same EAR-motif (IVLEM) (Figure 5a) while proteins A and B reported for common wheat contain the different EAR motifs IVLEM and KLHTLPNK, respectively, the latter shared with the barley Dro protein [59]. TtDro1A (346 aa) and TtDro1B (251 aa) share the C-terminal region with 8 differences, and in addition, TtDro1A has 96 extra amino acids in its N-terminal region (Figure 5a). The phylogenetic tree obtained (Figure 5b) shows that the TtDro1A and TtDro1B proteins cluster with those encoding the A and B subgenomes of common wheat. However, the TaDro1A protein sequence is more similar to the TtDro1A protein sequence of the *durum* subspecies than that of the *turgidum* subspecies, which could indicate a shorter evolutionary distance. There is also a higher similarity between the sequences of the proteins encoded by the *TaDDro1* gene of *T. aestivum* and *Ae. speltoides*, which is the donor of the D genome, with the TtDro1A protein than with the TtDro1B protein.

So far, all Dro-like proteins reported from different plant species are about 250 amino acids in size [59]. However, the protein encoded by the *TtDro1A* gene is 347 amino acids in size as the coding region is longer, which is supported by the recognition of the promoter consensus sequences. Identifying the promoter and transcription start site is not always easy, because many genes lack a TATA box and not all consensus sequences are easily identifiable. However, in this case, both promoters have a TATA box and the transcription factor B recognition element (BRE). In addition, the *TtDro1A* and *TtDro1B* introns are very different between both genes (Table S1), which indicates a long time of divergent sub-genomes evolution prior to the hybridisation process that gave rise to the allopolyploid. A mutation in the intron-exon region may have occurred in the parental donor of genome A, causing a change in the splicing process with the subsequent formation of a new protein. This difference was not previous reported by Ashraf et al. [59] when described the *Dro1* homeologues genes from *T. aestivum* subgenomes A, B and D.

In contrast with diploid plants, polyploids have more complex regulatory mechanisms to coordinate gene expression among homeologues and define their relative contribution to the phenotype [60]. The fact that the two genes *TtDro1A* and *TtDro1B* encode different proteins would be consistent with a distinct involvement in the signalling pathway to give steeper or less steep roots.

In order to find out whether there is a differential expression of the *TtDro1A* and *TtDro1B* genes between *durum* and *turgidum* subspecies, which could be involved in root angles development, we have carried out an expression study of these genes, considering four genotypes of each of the subspecies. The results obtained shown differences in all variables, except when comparing the expression of the *TtDro1A* gene in the primary root, in the seminal roots and in the mean of both types of roots (Figure 6).

When analysing the expression of *TtDro1A*, *TtDro1B* genes and their ratio taking into account the eight genotypes of *T. turgidum*, it was observed that there is difference in the expression of *TtDro1A* gene between the primary root and the seminal roots, with higher gene expression in the primary root (1.4 times) than in the seminal roots (Table 2). This result could indicate that this gene is involved in the difference in inclination between the two types of roots.

It should be noted that, in both subspecies and in both root types, the *TtDro1A* gene is expressed between 2.49 (AS/BS) to 8.76 (AP/BP) more than *TtDro1B* (Table 2, Figure 6), with clear statistically significant difference (Table 3). This agrees with the results obtained by Ashraf et al. [59] in common wheat that suggested that the presence of AuxRE motifs near the transcriptional start site in the *TaDro1B*-like promoter is the reason for the distinct expression of *DroA* and *DroB*. In the present work, the most noticeable difference between the *TtDro1B* genes of both subspecies is the presence of the element MITE in the 5′UTR region in the 4 genotypes of *turgidum* (data not shown) which leads us to think that this could be the cause of the lower expression of the gene in the *turgidum* subspecies. A similar finding was made by Xi et al. [61] who identified a MITE element in the 5′UTR region of the chalcone synthetase gene as responsible for the lower expression of the gene in the ‘Opata’ variety of *T. aestivum* relative to the expression of the same gene in the ‘Gy115′ variety without the insertion.

The *TtDro1A* and *TtDro1B* genes have differences in the nucleotide sequence, in the amino acid sequence of the proteins they encode and also differential expression, suggesting that they have different functions. However, our results indicate that both genes are involved in the root phenotype and specifically in root angle, which is supported by the high positive correlations obtained between the gene expression ratios of both root types and the corresponding angle data, while there was no correlation with the expression levels of *TtDro1A* and *TtDro1B* taken independently. (Table 4). Thus, although *TtDro1B* has a lower expression than *TtDro1A*, our data indicate that it plays an important role in the angle adopted by the roots. Therefore, the higher the expression of *TtDro1B* the lower the root angle, as observed in subspecies *durum* which has deeper roots than subspecies *turgidum* (Figure 2).

**Figure 7:**
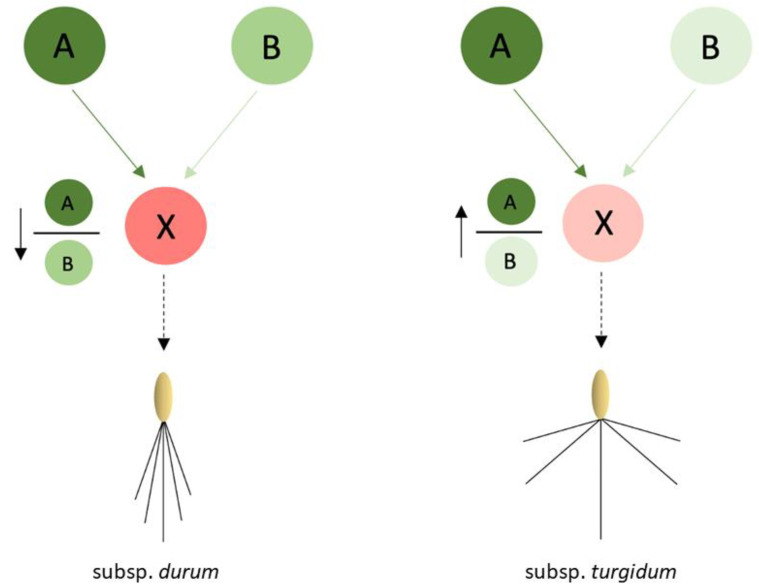
Representation of the involvement of *TtDro1A* (A) and *TtDro1B* (B) genes in a hypothetical signalling pathway responsible for root angle in *turgidum* and *durum* subspecies. Circle A represents *TtDro1A* expression. Circle B represents *TtDro1B* expression. The intensity of the colours is related to the expression of the genes. Circle X represents a hypothetical gene that would act downstream of *TtDro1A* and *TtDro1B* in the signalling pathway and the difference in colour would not be related to its level of expression. A more intense green colour in circle B indicates a higher expression of this gene and thus a lower ratio between *TtDro1A* and *TtDro1B* expression; a less intense green colour in circle B indicates a lower expression of *TtDro1B* and thus a higher ratio between *TtDro1A* and *TtDro1B* expression.

From the study carried out, we propose that root angle in durum wheat is determined, at least in part, by the differential expression of the *TtDro1A* and *TtDro1B* genes. Thus, the *TtDro1A* gene would be involved in the difference in inclination between the primary and the seminal roots, while the expression ratio between the two genes *TtDro1A*/*TtDro1B* is what determines whether the roots as a whole will be deeper as in *durum* subspecies or shallower as in *turgidum* subspecies, as shown in the hypothetical model of the Figure 7.

## 4. Materials and Methods

### 4.1. Plant Materials

In this work, eight genotypes of *durum* wheat (*Triticum turgidum* Desf.) belonging to the subspecies *durum* and *turgidum*, have been used. The materials were provided by the National Plant Genetic Resource Center of Spain (INIA-CRF), and their passport data are available at https://bancocrf.inia.es/en/ (accessed on 15 December 2020). (Table 5).

### 4.2. Methods

#### 4.2.1. Root Phenotyping

In this case, 12 seeds per genotype were disinfected with sodium hypochlorite solution (1.25%) during 15 min and rinsed 4 times with sterile distilled water. Seeds were placed in Petri dishes with of filter paper moistened with 4 mL of distilled water were kept at 4 °C for 2 days, and then put in the rhizoslide system according to the description of Ruiz et al. [47] and Boudiar et al. [62], and grown in a chamber at 22–18 °C with a photoperiod of 12 h of light for 1 week. After the seedling growth period, the roots were scanned at 300 ppi using a Canon “LiDE210” flat scanner. The images obtained from every seedling was analysed using the SmartRoot software v.3.32 [63] which is a plugin of ImageJ1.46R software (http://imagej.nih.gov/ij/download.html. Accessed 10 October 2019). For each seedling, the following variables were noted or calculated: number of roots (NR), total root length in cm (TRL), primary seminal root (hereinafter primary) length in cm (PRL), total root area in cm^2^ (S), total root volume in cm^3^ (V), mean root diameter in cm (DS), mean of root angles respect to the vertical in degrees (MRA), minimum root angle in degrees (MAV) and maximal root angle in degrees (MxRA). For statistical analysis, the 12 plantlets were divided into three replicates of four seedlings/replica.

#### 4.2.2. *TtDro1* Genes Genotyping

DNA of BGE 045630 and BGE048497 was extracted from leaves of 7-day-old seedlings, with the “NZY Plant/Fungi gDNA Isolation kit” (NZYTech, Lisbon, Portugal) following the instructions specified by the manufacturer. The specific primers for PCR amplification of the *Dro1* genes of *T. turgidum* were designed from the *T. aestivum* genomic sequences included in the Ensembl Plants database [48] which were homologous to the *Dro1* sequence of rice [20]. Given the large size of the candidate gene, 9 pairs of primers were manually designed (Table S1) to cover the entire gene regions with their PCR products overlapping, to obtain a unique and complete sequence of each gene.

#### 4.2.3. PCR Amplification and Fragment Cloning

PCR reactions were performed in a 25 μL volume containing 50 ng of genomic DNA using the primers designed for each subgenome with NZYTaq II 2× Green Master Mix (NZYTech, Lisbon, Portugal). The thermal cycling conditions for the amplification were: 94 °C for 5 min, followed by 38 cycles at 94 °C for 30 s, 55 °C for 30 s, 72 °C for 1.5 min, and a final extension of 72 °C for 10 min.

The fragments amplified by PCR were cloned with NZY-A PCR cloning kit (NZYTech), and 3 clones of each fragment were sequenced to rule out the Taq polymerase errors. All sequencing reactions was performed at the Molecular Biology Unit of the University of Alcalá (Alcalá de Henares, Spain). Sequence analysis were performed using software CodonCode Aligner v.3.7.1 (CodonCode Corporation, Centerville, MA, USA).

#### 4.2.4. Multiple Alignment Sequences and Phylogenetic Tree Construction

The alignment of the amino acid sequences of the Dro1 proteins was performed using the Clustal Omega multiple sequence alignment and MUSCLE 3.0 softwares, available at http://www.ebi.ac.uk/Tools/msa/clustalo/ (accessed 25 February 2022), on the EMBL-EBI website [64]. The Phylogeny.fr software was used to construct the phylogenetic tree [65].

#### 4.2.5. RT-qPCR

For the extraction of root RNA, 60 seeds/genotype were used and were germinated and grown in the same way as for the RSA study, but keeping them only for two days in the rhizoslide system, because that time is when the roots were observed to start developing and acquiring their growth angle.

For each genotype, RNA was extracted from the apical meristems of the primary seminal root and from the other seminal roots separately, using Tripure Reagent (Roche, Germany) according to the manufacturer’s instructions, and treated with Turbo RNase-free DNase (Ambion, Thermo Fisher Scientific, Whaltman, MA, USA) to remove all contaminating DNA. cDNA was synthesized from 1 μg of RNA employing the SMARTer PCR cDNA Synthesis Kit (Clontech, Takara Bio, Shiga, Japan) according to the manufacturer’s recommendations.

25 ng of cDNA from each sample were amplified in three technical replicates in a 7500 Fast Real Time PCR System Thermocycler (Thermo-Fisher Scientific, Whaltman, MA, USA), with primers designed from specific regions of *TtDro1A* and *TtDro1B* genes (Table S1). Ct values were determined and the relative expression level for each gene in the different templates calculated using the 2^−ΔΔCt^ method, normalized against the gene coding for malate dehydrogenase (MDH), which has been evaluated such as housekeeping gene in wheat by Pérez et al. [66]. The expression of the *TtDro1B* in primary roots from genotype BGE013733 was used as calibrator.

The following variables were calculated:

AP: expression of the *TtDro1A* gene in the primary root.
AS: expression of the *TtDro1A* gene in the seminal roots.
BP: expression of the *TtDro1B* gene in the primary root.
BS: expression of the *TtDro1B* gene in the seminal roots.
MA(P + S): average expression of the *TtDro1A* gene in the primary and seminal roots.
MB(P + S): average expression of the *TtDro1B* gene in the primary and seminal roots.
AP/BP: ratio between expressions of the genes *TtDro1A* and *TtDro1B* in the primary root.
AS/BS: ratio between expressions of the genes *TtDro1A* and *TtDro1B* in the seminal roots.
M(P + S)/MB(P + S): ratio between the average expressions of the genes *TtDro1A* and *TtDro1B* in the primary and the seminal roots.

#### 4.2.6. Statistical Analysis

Principal Component Analysis (PCA), *t*-Student, Fisher’s LSD test (Least Significant Difference), analysis of variance (ANOVA) and Pearson correlation were performed with the StatGraphics plus software v.5.1.

## 5. Conclusions

*Triticum turgidum* subspecies *durum* has a larger root system with more vertical roots than subspecies *turgidum*, which has shorter and shallower roots. The homeologous genes *TtDro1A* and *TtDro1B* show differences in their sequences, in the proteins they encode and in their expression levels in the roots of durum wheat seedlings. *TtDro1A* gene is expressed between 2.49 to 8.76 more than *TtDro1B* depending on the subspecies and type of seminal root. The *TtDro1A* gene is expressed more in the primary seminal root than in the other seminal roots. A positive and significant correlation has been observed between the root inclination angle and the expression ratio between the two genes. Thus, the lower this ratio (higher *TtDro1B* expression), the shallower the roots of durum wheat seedlings will be. This work demonstrates for the first time the relationship between the expression of *TtDro1* genes and the angle of inclination of the roots in durum wheat seedlings.

## Figures and Tables

**Table 1:** Means and standard deviation (SD) of the nine RSA variables in the eight genotypes analysed. The means ($\bar{X}$) of the four genotypes of each subspecies are highlighted in grey. (TRL) Total root length in cm, (PRL) primary root length in cm, (S) total root area in cm^2^; (V) total root volume in cm^3^; (DS) mean root diameter in cm; (NR) number of roots, (MRA) mean of root angles respect to the vertical in degrees, (MAV) minimum root angle in degrees and (MxRA) maximal root angle in degrees.

Genotype	Subsp.	TRL (SD)	PRL (SD)	S (SD)	V (SD)	DS (SD)	NR (SD)	MRA (SD)	MAV (SD)	MxAV (SD)
**BGE045630**	*durum*	108.57 (2.08)	25.36 (1.89)	17.16 (0.58)	0.225 (0.01)	0.051 (9 × 10^−4^)	5.58 (0.14)	15.43 (3.69)	5.67 (2.89)	27.42 (6.85)
**BGE018607**		115.88 (4.56)	24.96 (0.25)	19.06 (1.02)	0.25 (0.01)	0.05 (8 × 10^−4^)	5.58 (0.38)	15.91 (3.35)	6.90 (2.15)	26.66 (11.28)
**BGE045662**		101.85 (7.10)	24.62 (0.96)	18.56 (1.07)	0.275 (0.01)	0.05 (1 × 10^−3^)	5.16 (0.14)	16.13 (0.49)	7.08 (2.32)	31.33 (0.76)
**BGE045657**		115.62 (4.31)	28.04 (0.92)	19.87 (1.28)	0.28 (0.02)	0.054 (8 × 10^−4^)	5.58 (0.14)	23.42 (2.21	9.08 (0.80)	39.67 (3.98)
**$\bar{X}$*T. turgidum* subsp. *durum***		110.63 (9.63)	25.60 (1.64)	18.78 (0.95)	0.26 (0.02)	0.05 (2.8 × 10^−3^)	5.51 (0.23)	18.12 (3.56)	7.60 (1.01)	31.68 (5.65)
**BGE048497**	*turgidum*	63.70 (5.49	18.35 (0.97)	10.42 (1.14)	0.14 (0.01)	0.051 (2 × 10^−3^)	5 (0)	42.75 (1.96)	17.00 (1.32)	64.33 (2.42)
**BGE013733**		80.26 (3.18)	19.98 (0.77)	13.69 (0.22)	0.19 (0.002)	0.053 (1 × 10^−3^)	5.33 (0.14)	34.25 (2.98)	16.07 (6.81)	49.30 (4.51)
**BGE013081**		87.58 (0.29)	23.52 (1.25)	15.28 (0.34)	0.21 (0.01)	0.054 (1 × 10^−3^)	4.91 (0.14)	35.49 (0.92)	18.65 5.13)	53.80 (5.59)
**BGE018653**		68.44 (8.18)	19.13 (1.84)	11.79 (1.28)	0.16 (0.01)	0.053 (1 × 10^−3^)	5 (0.25)	33.55 (3.79)	14.26 (1.82)	50.57 (5.47)
**$\bar{X}$*T. turgidum* subsp. *turgidum***		76.38 (9.21)	20.79 (1.91)	13.15 (1.68)	0.19 (0.02)	0.05 (5.7 × 10^−4^)	5.06 (0.18)	36.52 (4.26)	16.74 (1.98)	54.36 (6.54)

**Table 2:** Means and standard deviation (SD) of the nine variables measuring *TtDro1A* and *TtDro1B* gene expression in the four genotypes of each of the subspecies and in the total of the 8 genotypes. AP, AS, BP and BS: expression of the *TtDro1A* and *TtDro1B* genes in the primary root (P) and the others seminal (S). MA(P + S): average expression of the *TtDro1A* gene in the primary root and the seminal roots. MB(P + S): average expression of the *TtDro1B* gene in the primary root and the seminal roots. AP/BP: ratio between expressions of the genes *TtDro1A* and *TtDro1B* in the primary root. AS/BS: ratio between expressions of the genes *TtDro1A* and *TtDro1B* in the seminal roots. M(P + S)/MB(P + S): ratio between the average expressions of the genes *TtDro1A* and *TtDro1B* in the primary and the seminal roots.

Variable	Subsp.		Both Subsp.(SD)
	*durum* (SD)	*turgidum* (SD)	
AP	9.51 (3.30)	10.19 (1.97)	9.85 (2.67)
AS	6.53 (2.73)	7.50 (1.97)	7.01 (2.38)
BP	3.87 (1.93)	1.24 (0.39)	2.55 (1.91)
BS	2.74 (1.31)	1.27 (0.21)	2.01 (1.18)
MA(P + S)	8.02 (2.96)	8.84 (1.55)	8.43 (2.34)
MB(P + S)	3.30 (1.59)	1.26 (0.24)	2.28 (1.52)
AP/BP	2.68 (0.76)	8.76 (2.36)	5.71 (3.54)
AS/BS	2.49 (0.51)	5.89 (1.27)	4.19 (1.98)
MA(P + S)/MB(P + S)	2.59 (0.63)	7.11 (0.95)	4.85 (2.43)

**Table 3:** Results of *t*-Student tests comparing the expression of the genes *TtDro1A* and *TtDro1B* in the primary the seminal roots and their ratio, considering the eight genotypes of the two *T. turgidum* subspecies. (-: *p* > 0.05; *: *p* < 0.05; ***: *p* < 0.005).

Variables	*p* Value
AP–AS	*
BP–BS	-
AP/BP–AS/BS	-
AP–BP	***
AS–BS	***
MA(P + S)-MB(P + S)	***

**Table 4:** Results of the Pearson correlations between the three variables of the RSA related with the angle of the roots, and the expression of the *TtDro1A*, the *TtDro1B* genes and their ratio in the primary and seminal roots of *T. turgidum*. (-: *p* > 0.05; **: 0.01 > *p* > 0.005; ***: *p* < 0.005).

Gene Expression	MAV (*p*)	Gene Expression	MxAV (*p*)	Gene Expression	MRA (*p*)
AP	r = 0.2626 (-)	AS	r = 0.3801 (-)	A(P + S)	r = 0.3108 (-)
BP	r = −0.6740 (-)	BS	r = −0.4927 (-)	B(P + S)	r = −0.6557 (-)
AP/BP	r = 0.9053 (***)	AS/BS	r = 0.8648 (**)	MA(P + S)/MB(P + S)	r = 0.9701 (***)

**Table 5:** Genotypes of *Triticum turgidum* subspecies used in this study.

Genotype Number	Passport Data	*T. turgidum* Subsp.
BGE045630 (abbreviated as 797)	https://bancocrf.inia.es/en/accessions/ESP004/BGE045630	*durum*
BGE018607	https://bancocrf.inia.es/en/accessions/ESP004/BGE018607	
BGE045662	https://bancocrf.inia.es/en/accessions/ESP004/BGE045662	
BGE045657	https://bancocrf.inia.es/en/accessions/ESP004/BGE045657	
BGE048497 (abbreviated as 869)	https://bancocrf.inia.es/en/accessions/ESP004/BGE048497	*turgidum*
BGE013733	https://bancocrf.inia.es/en/accessions/ESP004/BGE013733	
BGE013081	https://bancocrf.inia.es/en/accessions/ESP004/BGE013081	
BGE018653	https://bancocrf.inia.es/en/accessions/ESP004/BGE018653	

## Data Availability

The passport data of the plant material used in this work are available at https://bancocrf.inia.es/en/ (accessed on 15 December 2020) and in the Table 1. The sequences of the genes during the current study are available in GenBank with accession numbers MZ151530-MZ151533. The other data sets supporting the conclusions of this article are included within the article.

## Supplementary Materials

The following supporting information can be downloaded at: https://www.mdpi.com/article/10.3390/plants11060821/s1. Figure S1: Genomic sequence alignments of the *TtDro1A* and *TtDro1B* genes from durum wheat lines 797 (subspecies *durum*) and 869 (subspecies *turgidum*) by MUSCLE (3.8). Table S1: Primers used in PCR for *TtDro1* subgenomic amplifications and RT-qPCR analysis.
