# Supplementary material for: Isolation and Molecular Characterisation of TtDro1A and TtDro1B Genes from Triticum turgidum Subspecies durum and turgidum, Study of Their Influences on Seedling Root Angles
Source: Plants (Basel). 2022 Mar 19;11(6):821. doi: 10.3390/plants11060821 (PMC8954752; doi:10.3390/plants11060821)
Supplement: Supplementary file 1 [file plants-11-00821-s001.zip › plants-1601890-SI.pdf]

***DroA* sequences alignment**

```
DroA797       GCCGACCAGGCGCGATGGGATCCGTCGTGGCGCGGTCGTGACACTTGGTGCCCGGCCCTG
DroA869       GCCGACCAGGCGCGATGGGATCCGTCGTGGCGCGGTCGTGACACTTGGTGCCCGGCCCTG
              ************************************************************

DroA797       GGTTTGGGATCCGAAGGCCTATTATCTGTAGCCCATTTGGGTTAGTTTTCAGCTCGCAAC
DroA869       GGTTTGGGATCCGAAGGCCTATTATCTGTAGCCCATTTGGGTTAGTTTTCAGCTCGCAAC
              ************************************************************

DroA797       TTGGCTTGACATTATAGCTGCTTTTTAGGAGACGAACTGAAGTGGGTTCGCAAGGGGGTT
DroA869       TTGGCTTGACATTATAGCTGCTTTTTAGGAGACGAACTGAAGTGGGTTCGCAAGGGGGTT
              ************************************************************

DroA797       CCATTCTTTGGATCCCGCCCGGCGCAAAATCTCGATGGCCAAGTGACAAGAACGCCACCT
DroA869       CCATTCTTTGGATCCCGCCCGGCGCAAAATCTCGATGGCCAAGTGACAAGAACGCCACCT
              ************************************************************

DroA797       CTTCTCCCTGTTGAAAGAGCAACCGCGCCGAATACAGTCATCAATTGGTGTGCATGCAAA
DroA869       CTTCTCCCTGTTGAAAGAGCAACCGCGCCGAATACAGTCATCAATTGGTGTGCATGCAAA
              ************************************************************

DroA797       TTGCGGTACGTGCGTGTGATTTTATGTGTACGGGAGCATGTTAGATTTTTTCGACAAAGG
DroA869       TTGCGGTACGTGCGTGTGATTTTATGTGTACGGGAGCATGTTAGATTTTTTCGACAAAGG
              ************************************************************

DroA797       GGAGCACGTTAGATGCTGGTTTTTTTTCTTTCTCTAGAATACGCACGAGCATGCGTATCA
DroA869       GGAGCACGTTAGATGCTGGTTTTTTTTCTTTCTCTAGAATACGCACGAGCATGCGTATCA
              ************************************************************

DroA797       TATATTTATAGAAGAAGAGATGCTGGCTAAATTGCAATCTCTAAAAGAAGAGACCGGGTT
DroA869       TATATTTATAGAAGAAGAGATGCTGGCTAAATTGCAATCTCTAAAAGAAGAGACCGGGTT
              ************************************************************

DroA797       TATGAGATTGAGCAGTACTGATGTCAAAAAAAGGAGGGAATAGATAACACAGAAAGGTTT
DroA869       TATGAGATTGAGCAGTACTGATGTCAAAAAAAGGAGGGAATAGATAACACAGAAAGGTTT
              ************************************************************

DroA797       GTTCATCACATTCGCAAGGCCAGTAACAACACCACAGTTCAGAGCAATTATCCTCCCCGC
DroA869       GTTCATCACATTCGCAAGGCCAGTAACAACACCACAGTTCAGAGCAATTATCCTCCCCGC
              ************************************************************

DroA797       CGTATACAACTCCATGCGCCCCCTGTTTCAAATATAAAGCCTGGGCGTAGTTTCCAGACA
DroA869       CGTATACAACTCCATGCGCCCCCTGTTTCAAATATAAAGCCTGGGCGTAGTTTCCAGACA
              ************************************************************

DroA797       TCAATTGATGTGCTTGCAAATTGCAGGACGTGTGATTTTATATCGATTTGCAGTACGTGT
DroA869       TCAATTGATGTGCTTGCAAATTGCAGGACGTGTGATTTTATATCGATTTGCAGTACGTGT
              ************************************************************

DroA797       GGTTTTAGATAAACGCGATCATGTTGGATGCTGGCTAAATTTGCGTTGTACTACTTGCAA
DroA869       GGTTTTAGATAAACGCGATCATGTTGGATGCTGGCTAAATTTGCGTTGTACTACTTGCAA
              ************************************************************

DroA797       GATGCAACATGTTCAAAACTTGGGGAAATCGATCCAGACGGCGGGATCGAGAATAGTACT
DroA869       GATGCAACATGTTCAAAACTTGGGGAAATCGATCCAGACGGCGGGATCGAGAATAGTACT
              ************************************************************

DroA797       TGCTATTTTAGCTCAACACAGAGGTTACCATCTACGGCCTGGGCGTAGTTTACAGCGCCG
DroA869       TGCTATTTTAGCTCAACACAGAGGTTACCATCTACGGCCTGGGCGTAGTTTACAGCGCCG
              ************************************************************

DroA797       GCATGGAGTAGTTATGAGCTTATGACACTTGAGGCTGGATAGATAGAACTACCCACATGA
DroA869       GCATGGAGTAGTTATGAGCTTATGACACTTGAGGCTGGATAGATAGAACTACCCACATGA
              ************************************************************

DroA797       TAGCTCTAGGTACGATTCACACGGTCACAGTATCTTGATTACCTTAGAGTCGTCGTGCAT
```

```
DroA869         TAGCTCTAGGTACGATTCACACGGTCACAGTATCTTGATTACCTTAGAGTCGTCGTGCAT
                ************************************************************

DroA797         TTACGGCTTTACGCTGCACCGGTGACGGTTTTCAAGTTTAAACCGCGGGCACCGTAGTTG
DroA869         TTACGGCTTTACGCTGCACCGGTGACGGTTTTCAAGTTTAAACCGCGGGCACCGTAGTTG
                ************************************************************

DroA797         GGCTTTTCTAACCGATCCCCTGTTACTTGTTAAAAGAGTAAAAGTTTGGTTTACTTCTCT
DroA869         GGCTTTTCTAACCGATCCCCTGTTACTTGTTAAAAGAGTAAAAGTTTGGTTTACTTCTCT
                ************************************************************

DroA797         TAACGGCACCTAGCCGATCCCCAGTCGGTGTTAATGGAGAATAAATTATCCTCAACCGCG
DroA869         TAACGGCACCTAGCCGATCCCCAGTCGGTGTTAATGGAGAATAAATTATCCTCAACCGCG
                ************************************************************

DroA797         CCCCGTCTTCCCTATGTTCACTCCATCGCTCGGCGTTTGAGTAAAAAATCTCCTCTACTC
DroA869         CCCCGTCTTCCCTATGTTCACTCCATCGCTCGGCGTTTGAGTAAAAAATCTCCTCTACTC
                ************************************************************

DroA797         TGCTGCCACCTCCGCTCCACTCCCGCGCCACATCTACACTGGCGCCGCCCCTCCCACCCC
DroA869         TGCTGCCACCTCCGCTCCACTCCCGCGCCACATCTACACTGGCGCCGCCCCTCCCACCCC
                ************************************************************

DroA797         TGCTGGCCACCGCCTGCCACCAAGGACGCTCCGTAGGTTCCTCCGCGGTTCTTGACCACC
DroA869         TGCTGGCCACCGCCTGCCACCAAGGACGCTCCGTAGGTTCCTCCGCGGTTCTTGACCACC
                ************************************************************

DroA797         CGGATTTATGCCCTTCTCGCCCCGTCGCCAGCGCACAACCGGTAGATCTGCGGCTGCCGT
DroA869         CGGATTTATGCCCTTCTCGCCCCGTCGCCAGCGCACAACCGGTAGATCTGCGGCTGCCGT
                ************************************************************

DroA797         CGCCGCCGTCGGATTAGGCGCATCCAGCCGCCCGAGATTGCACCTTGACATGGATTGGCC
DroA869         CGCCGCCGTCGGATTAGGCGCATCCAGCCGCCCGAGATTGCACCTTGACATGGATTGGCC
                ************************************************************

DroA797         AGGTACCGTACCGCACAACCCCGGCAAAGTCTCTGCGCCGCATGCAGGTATTGACTCCAC
DroA869         AGGTACCGTACCGCACAACCCCGGCAAAGTCTCTGCGCCGCATGCAGGTATTGACTCCAC
                ************************************************************

DroA797         CTCTAGCGACTCGGATCTCTCCCGTCGGCGGTTCGCCGGCAAAGACACGCTCGGCCGTCG
DroA869         CTCTAGCGACTCGGATCTCTCCCGTCGGCGGTTCGCCGGCAAAGACACGCTCGGCCGTCG
                ************************************************************

DroA797         CTCGGGCTCGGCGCATTGCTGGTCATAAAGTGCGACGATTGCCCGCGACAGGTGGTGCGC
DroA869         CTCGGGCTCGGCGCATTGCTGGTCATAAAGTGCGACGATTGCCCGCGACAGGTGGTGCGC
                ************************************************************

DroA797         CGAGTTTCTACCACGCCAGAACATCCCGGATGGGTGTTCTTCAAGTGCAAAAATGATGGG
DroA869         CGAGTTTCTACCACGCCAGAACATCCCGGATGGGTGTTCTTCAAGTGCAAAAATGATGGG
                ************************************************************

DroA797         GTATGTGCTTCTTTTGACTCGGTTGTTCACCAGATTTGGCGCAATTTCGGTAGCTCATTC
DroA869         GTATGTGCTTCTTTTGACTCGGTTGTTCACCAGATTTGGCGCAATTTCGGTAGCTCATTC
                ************************************************************

DroA797         TTTGCTCAAATTTATGTGTAGGATGGATGCAAGTTTTGGTATTGGGAAGAAGATTACATC
DroA869         TTTGCTCAAATTTATGTGTAGGATGGATGCAAGTTTTGGTATTGGGAAGAAGATTACATC
                ************************************************************

DroA797         GATCTATTGATAGCATGGAATTTGATAGATGATCGTGCACTCGTTGCTAGAATAGAGGCT
DroA869         GATCTATTGATAGCATGGAATTTGATAGATGATCGTGCACTCGTTGCTAGAATAGAGGCT
                ************************************************************

DroA797         AGAGATGAGACTAGATACAGCAACGCCGAATTCGATGGAATCGAAGAAGAAAGAAGTGTA
DroA869         AGAGATGAGACTAGATACAGCAACGCCGAATTCGATGGAATCGAAGAAGAAAGAAGTGTA
                ************************************************************

DroA797         CAAACTCGAGAAGCCAATAGAGAGAGTCAACAATGAAGACATAGAGAACATATTAATCCA
```

```
DroA869         CAAACTCGAGAAGCCAATAGAGAGAGTCAACAATGAAGACATAGAGAACATATTAATCCA
                ************************************************************

DroA797         ACTAGTGGGAGCAGCTATGAAAGTTGGATATCTTTTAGAATGTCTAATTGTGGTTCTTAT
DroA869         ACTAGTGGGAGCAGCTATGAAAGTTGGATATCTTTTAGAATGTCTAATTGTGGTTCTTAT
                ************************************************************

DroA797         TTTCTTTGGTATTGCTCTTGTAGCAAAGAATTGGTGAATTATGTACTTCAACTTATTAAA
DroA869         TTTCTTTGGTATTGCTCTTGTAGCAAAGAATTGGTGAATTATGTACTTCAACTTATTAAA
                ************************************************************

DroA797         ATGTCGCTGAATGAAATTAAAAAGCAATAAATGTGTTTCTGTGGTTGAAAATGAAATGCA
DroA869         ATGTCGCTGAATGAAATTAAAAAGCAATAAATGTGTTTCTGTGGTTGAAAATGAAATGCA
                ************************************************************

DroA797         AATAGAAAAAAATTGCTTTGTTATATGTATGGGATCGGTTAGGTCCGGTGAAAACTTAGTG
DroA869         AATAGAAAAAAATTGCTTTGTTATATGTATGGGATCGGTTAGGTCCGGTGAAAACTTAGTG
                ************************************************************

DroA797         GGGTAAAAATACTTCACTAAGGGTTTACTTCGTCGGATTCTTCGTTATTTTTAGAGGGTC
DroA869         GGGTAAAAATACTTCACTAAGGGTTTACTTCGTCGGATTCTTCGTTATTTTTAGAGGGTC
                ************************************************************

DroA797         GGTTAGAAATGCGCTTAGTTCGCACCTTTTTACAACGAGAGTAGAGTTACTTTTGCACTT
DroA869         GGTTAGAAATGCGCTTAGTTCGCACCTTTTTACAACGAGAGTAGAGTTACTTTTGCACTT
                ************************************************************

DroA797         GAAGGCTAATAATAGCGTGGACGTACATGCATATCATTTCGACTGTAAAATGTAAATTTC
DroA869         GAAGGCTAATAATAGCGTGGACGTACATGCATATCATTTCGACTGTAAAATGTAAATTTC
                ************************************************************

DroA797         TCTCGAGAAATGTAGATCGACAGGCTGAGATTTTTTTAGCCGGTAATGATAAACTCACGT
DroA869         TCTCGAGAAATGTAGATCGACAGGCTGAGATTTTTTTAGCCGGTAATGATAAACTCACGT
                ************************************************************

DroA797         CTGGCTGGCTGGCTGGCGCCATCTCATAATGCTCGTCCCTCCCAAAAGGGAAGGTCACCA
DroA869         CTGGCTGGCTGGCTGGCGCCATCTCATAATGCTCGTCCCTCCCAAAAGGGAAGGTCACCA
                ************************************************************

DroA797         TCTCGGAGAAAAATAAGGCCGGGAAGTGACATGCCAGATGAAGCATGTATAGCTAGCGGC
DroA869         TCTCGGAGAAAAATAAGGCCGGGAAGTGACATGCCAGATGAAGCATGTATAGCTAGCGGC
                ************************************************************

DroA797         ACCTCATGAGCCACGCTACAAAGCCTTGGGACTTGAGCCACCCCGGCCCCTTCAAATCAA
DroA869         ACCTCATGAGCCACGCTACAAAGCCTTGGGACTTGAGCCACCCCGGCCCCTTCAAATCAA
                ************************************************************

DroA797         ATCGCTCCAATAAGCAAGCCGGGCTACCGGTAGTAGCTCCACACTGCCACTCACTGCGCG
DroA869         ATCGCTCCAATAAGCAAGCCGGGCTACCGGTAGTAGCTCCACACTGCCACTCACTGCGCG
                ************************************************************

DroA797         ACACCGCTCCTTCCATCGCCGGTAGCAGCAGCCACCACCACCACTTCAAGTGCAAGGCCT
DroA869         ACACCGCTCCTTCCATCGCCGGTAGCAGCAGCCACCACCACCACTTCAAGTGCAAGGCCT
                ************************************************************

DroA797         CTCACACCCTCTCTCAGTCATCAGCTCCTCATCACTCTGCTTCTTGCAGTCTCGGCACCT
DroA869         CTCACACCCTCTCTCAGTCATCAGCTCCTCATCACTCTGCTTCTTGCAGTCTCGGCACCT
                ************************************************************

DroA797         GCATCGGCTTCGCCGCTCATTCCCACATCCCGATCAATCCCCAAGCAAGGAAGATGAAGG
DroA869         GCATCGGCTTCGCCGCTCATTCCCACATCCCGATCAATCCCCAAGCAAGGAAGATGAAGG
                ************************************************************

DroA797         TAAAGGCCCATGCCATCGCCCACCCCTGTGATCTTTATTTTTGCAGTAGGTTTGATGTGC
DroA869         TAAAGGCCCATGCCATCGCCCACCCCTGTGATCTTTATTTTTGCAGTAGGTTTGATGTGC
                ************************************************************

DroA797         TTGAATGTCTGAATGGATAGTGGCTGCTGCTGCTGATGATGATGATGATGGAACCTCTGT
```

```
DroA869        TTGAATGTCTGAATGGATAGTGGCTGCTGCTGCTGATGATGATGATGATGGAACCTCTGT
               ************************************************************

DroA797        TTGCGTGGTGGCGTTCTGGTGAAGTGACAGAACTGGCCTCTGTTTTTCTTGCAGATTTTC
DroA869        TTGCGTGGTGGCGTTCTGGTGAAGTGACAGAACTGGCCTCTGTTTTTCTTGCAGATTTTC
               ************************************************************

DroA797        AGCTGGATGGCGAACAAGATCAGCGGGAAGCAGGAAGCGAGCCGCTTCCCGGCCAGTTCC
DroA869        AGCTGGATGGCGAACAAGATCAGCGGGAAGCAGGAAGCGAGCCGCTTCCCGGCCAGTTCC
               ************************************************************

DroA797        TCGGGCCCTTCTCGTAAGTGCACTTTGGAACCTTCGCTTTCTTGTTGACCAAATAAGTAG
DroA869        TCGGGCCCTTCTCGTAAGTGCACTTTGGAACCTTCGCTTTCTTGTTGACCAAATAAGTAG
               ************************************************************

DroA797        CGTGTGCTCTAGACTAGTGTAGAAGCAAAAT-GGTCATCATTTCCCTTGATTCCCTGAAA
DroA869        CGTGTGCTCTAGACTAGTGTAGAAGCAAAATGGGTCATCATTTCCCTTGATTCCCTGAAA
               ******************************* ****************************

DroA797        AGATGAGGAAGAAGGCGGTCATCTTTGATCTCTGCTTATAATCCTTGACAAAAAAAAAAT
DroA869        AGATGAGGAAGAAGGCGGTCATCTTTGATCTCTGCTTATAATCCTTGACAAAAAAAAAAT
               ************************************************************

DroA797        ATCATTTTTGCCTTCAAGTTTCACTTGTGTCAGTTAGCTCTGGTCATCAAAGTTATTTTT
DroA869        ATCATTTTTGCCTTCAAGTTTCACTTGTGTCAGTTAGCTCTGGTCATCAAAGTTATTTTT
               ************************************************************

DroA797        TTGCTGCCTCTCCCAGATGAAATTAAAATGATGAAGTGGA-TTTGCTGGTCCCTCCCCAC
DroA869        TTGCTGCCTCTCCCAGATGAAATTAAAATGATGAAGTGGATTTTGCTGGTCCCTCCCCAC
               **************************************** *******************

DroA797        TTCCGGTCTTGTTTTTTTTAACGATTCTGGGATTTCTTCTAACCCTTTCGCTCTGGGATT
DroA869        TTCCGGTCTTGTTTTTTTTAACGATTCTGGGATTTCTTCTAACCCTTTCGCTCTGGGATT
               ************************************************************

DroA797        TTCCGCAATCCGTTAGTCTTTAGATCTATTGGTCTAGTGTGGAAGCTACAGCCTCTATAA
DroA869        TTCCGCAATCCGTTAGTCTTTAGATCTATTGGTCTAGTGTGGAAGCTACAGCCTCTATAA
               ************************************************************

DroA797        CTTAATATAAGACTTTTTTTTTACTCTAAAACCGTCTTATAAAAAGTTACAGAGGGAGTA
DroA869        CTTAATATAAGACTTTTTTTTTACTCTAAAACCGTCTTATAAAAAGTTACAGAGGGAGTA
               ************************************************************

DroA797        CCTTTCACGACTTATGATAATGCCAGTGTACACCGTCAAAGAAAATGCCAGTGCAGTGAC
DroA869        CCTTTCACGACTTATGATAATGCCAGTGTACACCGTCAAAGAAAATGCCAGTGCAGTGAC
               ************************************************************

DroA797        ATTCCCTGACTTGCCTGCAATTTTTTTACTGTTTGTTTTCTTTAATGGACTTGTGTATCA
DroA869        ATTCCCTGACTTGCCTGCAATTTTTTTACTGTTTGTTTTCTTTAATGGACTTGTGTATCA
               ************************************************************

DroA797        AGGGGTTTCCTCACTCTTTTCTATCCCTAGCTCTTTTTCATGGTTTCCTGTAAGGTTTTC
DroA869        AGGGGTTTCCTCACTCTTTTCTATCCCTAGCTCTTTTTCATGGTTTCCTGTAAGGTTTTC
               ************************************************************

DroA797        CGCCCAGCTAGTCGTCATGGATCAAAGCCTGAACTAGTGGAAGCAAGTACATTTTTGTTC
DroA869        CGCCCAGCTAGTCGTCATGGATCAAAGCCTGAACTAGTGGAAGCAAGTACATTTTTGTTC
               ************************************************************

DroA797        CTACATACTTTTGATTTCCTCACGATTGTTGTTCTTTATGTAAGAAATGCCATGATTACT
DroA869        CTACATACTTTTGATTTCCTCACGATTGTTGTTCTTTATGTAAGAAATGCCATGATTACT
               ************************************************************

DroA797        TTATAGCCCCAACCCCCAACCTGTCAACAAAAATAATGTCATTCATTCTGGGTAGAATCC
DroA869        TTATAGCCCCAACCCCCAACCTGTCAACAAAAATAATGTCATTCATTCTGGGTAGAATCC
               ************************************************************

DroA797        TGACACCTTGGATGGTTTTGTTCCAATGAGCTGAAGTCAAGCAATCCTGTTTAAACATAT
```

```
DroA869         TGACACCTTGGATGGTTTTGTTCCAATGAGCTGAAGTCAAGCAATCCTGTTTAAACATAT
                ************************************************************

DroA797         TCCGTGTCAAGAAGACATAAAAAAAAGGCTACTGATATTGCAGAATGGATGCTCTATTTAA
DroA869         TCCGTGTCAAGAAGACATAAAAAAAAGGCTACTGATATTGCAGAATGGATGCTCTATTTAA
                ************************************************************

DroA797         CTTTTTATAGATGTGATCAAGTGATTGCCGACATACAAAAAATGAAAGAGAACATCAGGA
DroA869         CTTTTTATAGATGTGATCAAGTGATTGCCGACATACAAAAAATGAAAGAGAACATCAGGA
                ************************************************************

DroA797         TGTGGAGTAGGCATGGGCAAAAACATATATCTAACCAGTGATAAGCTGATGAGAATGACT
DroA869         TGTGGAGTAGGCATGGGCAAAAACATATATCTAACCAGTGATAAGCTGATGAGAATGACT
                ************************************************************

DroA797         GGGGCTTAAGAATTTTAAACTTAGTGGTCCATGCTCCGGACACCCAATTTAAGCTAGTAT
DroA869         GGGGCTTAAGAATTTTAAACTTAGTGGTCCATGCTCCGGACACCCAATTTAAGCTAGTAT
                ************************************************************

DroA797         GAACTTAGGACCATACCTCTATGTCTTTTCATGGAACTATCAACTTAATTTAGTTCTAC
DroA869         GAACTTAGGACCATACCTCTATGTCTTTTCATGGAACTATCAACTTAATTTAGTTCTAAT
                ************************************************************

DroA797         GTGCAGGTTCTAACGTGCCGGATTGTCGCAACGACGAGTTCAGCGATTGGCCCCAATCAT
DroA869         GTGCAGGTTCTAACGTGCCGGATTGTCGCAACGACGAGTTCAGCGATTGGCCCCAATCAT
                ************************************************************

DroA797         TGCTTGCCATTGGGACATTTGGAAATAAGCAGATAGAGGAGGTAGCACAAGTGCAGGACA
DroA869         TGCTTGCCATTGGGACATTTGGAAATAAGCAGATAGAGGAGGTAGCACAAGTGCAGGACA
                ************************************************************

DroA797         CTTCCGAGGATGGGCAGTCCATGCAAGATGCCATCAAGTTTACAGAGGAGGAAGTAGACA
DroA869         CTTCCGAGGATGGGCAGTCCATGCAAGATGCCATCAAGTTTACAGAGGAGGAAGTAGACA
                ************************************************************

DroA797         GGATACAGAAAGAGTTTGCAATGATACTAGCAAGCAAAGACCAAGCAGAAGCCCATGACT
DroA869         GGATACAGAAAGAGTTTGCAATGATACTAGCAAGCAAAGACCAAGCAGAAGCCCATGACT
                ************************************************************

DroA797         CGCACGATGATGAGCAGGTAACTTCACACAAAGAAGTCGATGAGAGCATAAATGAGAAGC
DroA869         CGCACGATGATGAGCAGGTAACTTCACACAAAGAAGTCGATGAGAGCATAAATGAGAAGC
                ************************************************************

DroA797         ACAGGGACCAGCTGCTGAACAAGATGGTCGTCATAAGTAAGGCAAAAGATTCACTGGGGA
DroA869         ACAGGGACCAGCTGCTGAACAAGATGGTCGTCATAAGTAAGGCAAAAGATTCACTGGGGA
                ************************************************************

DroA797         AGAAAGCAAGTACACTTAAGCCGAGGTCGGTTGCTTCGCTCTTCAAACTACTCATGCGCA
DroA869         AGAAAGCAAGTACACTTAAGCCGAGGTCGGTTGCTTCGCTCTTCAAACTACTCATGCGCA
                ************************************************************

DroA797         AAGGTGGCTTTGCCTCTGCTATTCCAGATCCAAGGAGCTCTTTCCCTCAATCAAGAATGG
DroA869         AAGGTGGCTTTGCCTCTGCTATTCCAGATCCAAGGAGCTCTTTCCCTCAATCAAGAATGG
                ************************************************************

DroA797         AAAAGGTTCACTTACATTCTTATGTACATCTACAGTTAATTATACCTTCTTGACTCTTCA
DroA869         AAAAGGTTCACTTACATTCTTATGTACATCTACAGTTAATTATACCTTCTTGACTCTTCA
                ************************************************************

DroA797         GTAGTATCTTATGGTTTGATTTTCTTCCCATTTCAGCTGCTCAAGGCAATACTTCAGAAG
DroA869         GTAGTATCTTATGGTTTGATTTTCTTCCCATTTCAGCTGCTCAAGGCAATACTTCAGAAG
                ************************************************************

DroA797         AAAATACACCCGCAAAATTCTTCGACGCTCGTACCTAGGAGACATTTGGACTGGAAGCCG
DroA869         AAAATACACCCGCAAAATTCTTCGACGCTCGTACCTAGGAGACATTTGGACTGGAAGCCG
                ************************************************************

DroA797         GATGAGCAAGAGATCAATGAATGCCTCGAGGATGCACTCCGTGATCTAGACGACGATGGC
```

```
DroA869          GATGAGCAAGAGATCAATGAATGCCTCGAGGATGCACTCCGTGATCTAGACGACGATGGC
                 ************************************************************

DroA797          GCAAAATGGGTCAAAACTGATTCAGACTGTAAGTTGCATACTTTGATCCCGAACAAGTGG
DroA869          GCAAAATGGGTCAAAACTGATTCAGACTGTAAGTTGCATACTTTGATCCCGAACAAGTGG
                 ************************************************************

DroA797          CGTTATCGTGCACTGAACATCTCTTTATGCACTGATTCCGTCAAAGAAACTCACTATGTT
DroA869          CGTTATCGTGCACTGAACATCTCTTTATGCACTGATTCCGTCAAAGAAACTCACTATGTT
                 ************************************************************

DroA797          ATTTTCATTCTTTGCAGTTATTGTGCTAGAAATGTAAACATGGGTGAATACATTTGTTCA
DroA869          ATTTTCATTCTTTGCAGTTATTGTGCTAGAAATGTAAACATGGGTGAATACATTTGTTCA
                 ************************************************************

DroA797          TTGAGGCAGGTGTGCATCTTTTTTTTTTCTCAATTCACGACTATCAGGAGAACTCATTGC
DroA869          TTGAGGCAGGTGTGCATCTTTTTTTTTTCTCAATTCACGACTATCAGGAGAACTCATTGC
                 ************************************************************

DroA797          TAATATTTATCCTTTATGTTGAGTGTTGTGCTTGCAATTTGATTGATTACAATGTGCAGG
DroA869          TAATATTTATCCTTTATGTTGAGTGTTGTGCTTGCAATTTGATTGATTACAATGTGCAGG
                 ************************************************************

DroA797          TTCCAAAGATCTTGGCAAGGTAGGGATCCGCGGTGTTATGATATATATATAGAGGTGTGG
DroA869          TTCCAAAGATCTTGGCAAGGTAGGGATCCGCGGTGTTATGATATATATATAGAGGTGTGG
                 ************************************************************

DroA797          GATATAGTCATCAATGAAGCTACGAATTATGATGCAATGCCCCTCTGTTTCTCATATAAA
DroA869          GATATAGTCATCAATGAAGCTACGAATTATGATGCAATGCCCCTCTGTTTCTCATATAAA
                 ************************************************************

DroA797          TCATTTGTAATTGGGTTACTGGGATTATGATAAGGAACTACTGGGACAAGCCATACCTCA
DroA869          TCATTTGTAATTGGGTTACTGGGATTATGATAAGGAACTACTGGGACAAGCCATACCTCA
                 ************************************************************

DroA797          TGTCCGTGTTTTTCTCTAGCCCTTCAGACTGTACATTACAAGGTCTTTTACTATTCACAT
DroA869          TGTCCGTGTTTTTCTCTAGCCCTTCAGACTGTACATTACAAGGTCTTTTACTATTCACAT
                 ************************************************************

DroA797          GAACAATGATGATGGATGTTTCTGTCCTCCTTAAATGAATTCAATTATGATACAGTTGTT
DroA869          GAACAATGATGATGGATGTTTCTGTCCTCCTTAAATGAATTCAATTATGATACAGTTGTT
                 ************************************************************

DroA797          ATTTTATCACATATATTTTCAATTTAGATCAGCTTTCAAGTTGTGCCCAGTAGATCTGGG
DroA869          ATTTTATCACATATATTTTCAATTTAGATCAGCTTTCAAGTTGTGCCCAGTAGATCTGGG
                 ************************************************************

DroA797          TTTCATGCCAGAAAGTTAAGATCCTCTTTGATCTGTAGGATTTGAAGAAGACACTAATAT
DroA869          TTTCATGCCAGAAAGTTAAGATCCTCTTTGATCTGTAGGATTTGAAGAAGACACTAATAT
                 ************************************************************

DroA797          AATAAATACAAGATTGTAAAGGCATGCCCTTTTTGATCTTCCACGATTAGAGAACA
DroA869          AATAAATACAAGATTGTAAAGGCATGCCCTTTTTGATCTTCCACGATTAGAGAACA
                 ********************************************************
```

***DroB* sequences alignment**

```
DroB797          CGGGCAAGTAACATACCGATTGACAAAGAGAATTATGTATGTTGTCATAACGGTTCG-CC
DroB869          CGGGCAAGTAACATACCGATTGACAAAGAGAATTATGTATGTTGTCATAACGGTTCGACC
                 ********************************************************* **

DroB797          GATAAAGATCTTCGTAGAATATGTGGAATCCAATATGAACATCCAGGTTCCGCTATTGGT
DroB869          GATAAAGATCTTCGTAGAATATGTGGAATCCAATATGAACATCCAGGTTCCGCTATTGGT
                 ************************************************************

DroB797          TATTGACCGGAGAGGTGTCTTGGTCATGTCTACATAGTTCTCGAACCCGTAGGGTCCGCA
```

```
DroB869         TATTGACCGGAGAGGTGTCTTGGTCATGTCTACATAGTTCTCGAACCCGTAGGGTCCGCA
                ************************************************************

DroB797         CGCTTAACGTTCGTCGACAATATAGTGTTATATGAGTTATATGATTTGGTGACTGAATGT
DroB869         CGCTTAACGTTCGTCGACAATATAGTGTTATATGAGTTATATGATTTGGTGACTGAATGT
                ************************************************************

DroB797         TGTTCAGAGTCCCGGATGAGATCACAGACATGACGAGGAGTCTCGAAATGGTCGAGAGGT
DroB869         TGTTCAGAGTCCCGGATGAGATCACAGACATGACGAGGAGTCTCGAAATGGTCGAGAGGT
                ************************************************************

DroB797         AAAGATTGATATATATAGGACGATGCTATTCGGACACCGGAAGAGTTTCGGAGTGAACCG
DroB869         AAAGATTGATATATATAGGACGATGCTATTCGGACACCGGAAGAGTTTCGGAGTGAACCG
                ************************************************************

DroB797         GGTAACCATCGGGTCACCGAAAGGGGGAGGGGGTGTTGAGTATATAGTGCATGTATATTT
DroB869         GGTAACCATCGGGTCACCGAAAGGGGGAGGGGGTGTTGAGTATATAGTGCATGTATATTT
                ************************************************************

DroB797         GTATTTCCGGCCGACCTCCTTTTACCTTGTATAGTTGAGGTTGTGGCCCACCTTGTACAT
DroB869         GTATTTCCGGCCGACCTCCTTTTACCTTGTATAGTTGAGGTTGTGGCCCACCTTGTACAT
                ************************************************************

DroB797         CATATATATGTGCGCTTGTACCCGATTAATACATGGAGAGTCGCATCCTATTCCTCTACA
DroB869         CATATATATGTGCGCTTGTACCCGATTAATACATGGAGAGTCGCATCCTATTCCTCTACA
                ************************************************************

DroB797         AGCCGGACGCTCCAAATCCGCCTCAAACGTCTTGGTGGACGACCCGGTCACTGATCGGTC
DroB869         AGCCGGACGCTCCAAATCCGCCTCAAACGTCTTGGTGGACGACCCGGTCACTGATCGGTC
                ************************************************************

DroB797         ACGAAAATTCGACCTAGATGGGCTCCTCAAACTTGTCTCAAACGTCCGGGCTGACTGGCA
DroB869         ACGAAAATTCGACCTAGATGGGCTCCTCAAACGTGTCTCAAACGTCCGGGCTGACTGGCA
                ******************************** ***************************

DroB797         GCCCGCATATCCAGCCCAAATATGGGACGAATATGGGGGCGCCCGGGCACGTCCTCAGAC
DroB869         GCCCGCATATCCAGCCCAAATATGGGACGGATATGGGGGCGCCCGGGCACGTCCTCAGAC
                ***************************** ******************************

DroB797         CCCGTTGCAGGTCGAAGTGGTCCCACCTGCAGCGACAGAAACTCCTTCCTCTCTCATCGG
DroB869         CCCGTTGCAGGTCGAAGTGGTCCCACCTGCAGCGACAGAAACTCCTTCCTCTCTCATCGG
                ************************************************************

DroB797         CGCACCCAAATTTTAGTCCCACATCACCACGCGAGGCGGATGCCAACCGACTTAAATAGC
DroB869         CGCACCCAAATTTTAGTCCCACATCACCACGCAAGGCGGATGCCAACCGACTTAAATAGC
                ******************************** ***************************

DroB797         CGCACCTGCGGATGGCGGCATGCTCCTCTGTTGTTGTGTAGGTCTATCAATTCTCGCTGT
DroB869         CGCACCTGCGGATGGCGGCATGCTCCTCTGTTGTTGTGTAGGTCTATCAATTCTCGCTGT
                ************************************************************

DroB797         TTGAACAAGATAACATGACACTAGACTGCATTTCATCAACTGATAATTGTTCATCGAGTG
DroB869         TTGAACAAGATAACATGACACTAGACTGCATTTCATCAACTGATAATTGTTCATCGAGTG
                ************************************************************

DroB797         TCAAGTTCGTCTCGTTCATGTACATTGATGCCCCTACTACAATGAGAGTAGATTTTTATT
DroB869         TCAAGTTCGTCTCGTTCATGTACATTGATGCCCCTACTACAATGAGAGTAGATTTTTATT
                ************************************************************

DroB797         TTGTTCATCGATTTTTATCGACAGATTGTGGTTCATAAAAAATAGTATTGACATGAATGG
DroB869         TTGTTCATCGATTTTTATCGACAGATTGTGGTTCATAAAAAATAGTATTGACATGAATGG
                ************************************************************

DroB797         TTCTCTTGTCAAGGAGATAGACCCTGACTATGAATCTATCTTCTTAACAAAGGATGCAAT
DroB869         TTCTCTTGTCAAGGAGATAGACCCTGACTATGAATCTATCTTCTTAACAAAGGATGCAAT
                ************************************************************

DroB797         GATGTCAGAAGCTTATCGCTTTTGGACAACCCGGCTATTTAAGTCGGTCGACTAGTTTTT
```

```
DroB869         GATGTCAAAAGCTTATCGCTTTTGGACAACCCGGCTATTTAAGTCGGTCGACTAGTTTTT
                ******* ****************************************************

DroB797         CACACGGCAAGGTGTGACTAATACAAGTGTGAAAATCTTAAGTATGAGCAATTTTTGTAT
DroB869         CACGCGGCAAGGTGGGACTAATACAAGTGTGAAAATCTTAAGTATGAGCAAATTTTGTAT
                *** ********** ************************************ ********

DroB797         GTTTTGACGGTCCAGCCGGCCCAAGACGGACGGAATGATCGTTGTGCATGGCTCATGTAA
DroB869         GTTTTGACGGTCCAGCCGGCCCAAGACGGACGGAATGATCGTTGTGCATGGCTCATGTAA
                ************************************************************

DroB797         TAAATGTTCTAAAAATAGTGTATGTGGTTGTAAATGGTAAAATTTGAGGCCCGCCCGCGT
DroB869         TAAATGTTCTAAAAATAGTGTATGTGGTTGTAAATGGTAAAATTTGAGGCCCGCACGCGT
                ****************************************************** *****

DroB797         CCGCGGACAGATCATTTCGAATGCATATTTCTCTGGAGAAATATCGATCGACCAGCTGAT
DroB869         CCGCGGACAGATCATTTCGAATGCATATTTCTCTGGAGAAATATCGATCGACCAGCTGAT
                ************************************************************

DroB797         ACGGGGGAGATTTTCTTGAGGTGCTAAAACCCACGTCTGGCTGGCTGGCGCCACCTCAGA
DroB869         ACGGGGGAGATTTTCTTGAGGTGCTAAAACCCACGTCTGGCTGGCTGGCGCCACCTCAGA
                ************************************************************

DroB797         ATGCTCCTCCGAAAAGGGAATGTCAACATCTCGGAGAAAAATAAGGCCAGGAAGTGACAT
DroB869         ATGCTCCTCCGAAAAGGGAATGTCAACATCTCGGAGAAAAATAAGGCCAGGAAGTGACAT
                ************************************************************

DroB797         GCCAGATGAAGCATGTATAAGCTA------------------------------------
DroB869         GCCAGATGAAGCATGTATAAGCTACTCCCTCCGTTTCAAAATAGATGACCCAATTTTATA
                ************************

DroB797         --GCTAGCAGCACCTCATCAGCCACGCTACAAAGCCTTGGGACTTGCGCCACCCCCTTCA
DroB869         CGGCTAGCAGCACCTCATCAGCCACGCTACAAAGCCTTGGGACTTGCGCCACCCCCTTCA
                  **********************************************************

DroB797         AATCGCTCCAATAAGCAAGCCGAGCTACTAGTAGTAGCTCCACACTGCCACTCACTGTGT
DroB869         AATCGCTCCAATAAGCAAGCCGAGCTACTAGTAGTAGCTCCACACTGCCACTCACTGTGT
                ************************************************************

DroB797         GACACCGCTTCTTCCATCGCCAGTAGCAGCGGCCACCACCACCGCTTCAAGTGCAAGGCC
DroB869         GACACCGCTTCTTCCATCGCCAGTAGCAGCGGCCACCACCACCGCTTCAAGTGCAAGGCC
                ************************************************************

DroB797         TCTCGCCTACATCTCTCGCACTCTCTCTCGGTCATCAGCTCCTCATCACTCTGCCTCTTG
DroB869         TCTCGCCTACATCTCTCGCACTCTCTCTCGGTCATCAGCTCCTCATCACTCTGCCTCTTG
                ************************************************************

DroB797         CAGTCTCGGCACCCGCATCGGCTTCGCCGCTCACTCCCGACCTCCATTCCTAAGCAAGGA
DroB869         CAGTCTCGGCACCCGCATCGGCTTCGCCGCTCACTCCCGACCTCCATTCCTAAGCAAGGA
                ************************************************************

DroB797         AGATGAAGGTAAAGGCTCATGCCATCGCCCACCGATGCGATCTTTATTTTGCAGTAGGTT
DroB869         AGATGAAGGTAAAGGCTCATGCCATCGCCCACCGATGCGATCTTTATTTTGCAGTAGGTT
                ************************************************************

DroB797         TCATGTGCTTGAAAAGGTGCGGATGGGTGGTTGACTGGTGATGGCGGAACCTCTGCTTGC
DroB869         TCATGTGCTTGAAAAGGTGCGGATGGGTGGTTGACTGGTGATGGCGGAACCTCTGCTTGC
                ************************************************************

DroB797         GTGGTGGCGCTATGGTAATGTGACCAACCTCTGTTTTTCTTGCAGATTTTCAGCTGGATG
DroB869         GTGGTGGCGCTATGGTAATGTGACCAACCTCTGTTTTTCTTGCAGATTTTCAGCTGGATG
                ************************************************************

DroB797         GCGAACAAGATCAGCGGGAAGCAGGAGGCCAGCCGCTTCCCGGCCAGTTCCTCCGGGCCT
DroB869         GCGAACAAGATCAGCGGGAAGCAGGAGGCCAGCCGCTTCCCGGCCAGTTCCTCCGGGCCT
                ************************************************************

DroB797         TCTCGTGAGTGCGCTTGGGAAGAACTTCCGCTTTCTTGTTCATCAAATAAGTACTGTGTG
```

```
DroB869         TCTCGTGAGTGCGCTTGGGAAGAACTTCCGCTTTCTTGTTCATCAAATAAGTACTGTGTG
                ************************************************************

DroB797         CTATAGACTAGTCCAGAAGAACGACCACCATTTCCTTTAACCGCTTGAAAACATGAGGAA
DroB869         CTATAGACTAGTCCAGAAGAACGACCACCATTTCCTTTAACCGCTTGAAAACATGAGGAA
                ************************************************************

DroB797         AAAGACAGTATCTTTGTTCTCTGCTTTTACTCCTTGAAAAATGGTTACATCTTTGCTCCG
DroB869         AAAGACAGTATCTTTGTTCTCTGCTTTTACTCCTTGAAAAATGGTTACATCTTTGCTCCG
                ************************************************************

DroB797         AAGTTTCACCTGCGTCGGTTAGCTCCGGTCATCAAAGTTATTTTTGCTTCCTCGGCCCAA
DroB869         AAGTTTCACCTGCGTCGGTTAGCTCCGGTCATCAAAGTTATTTTTGCTTCCTCGGCCCAA
                ************************************************************

DroB797         AATAAAATAAAATTGATGAAGTAATTTTGCTTGTCCCTCCCCCTGCTGGTCTTT-TTTTT
DroB869         AATAAAATAAAATTGATGAAGTAATTTTGCTTGTCCCTCCCCCTGCTGGTCTTTCTTTTT
                ****************************************************** *****

DroB797         TAATGATTCAAGGATCTCTAATTTTCCGCACTCCGTTGGTCTTTAGATCTAGTAATGGTC
DroB869         TAATGATTCAAGGATCTCTAATTTTCCGCACTCCGTTGGTCTTTAGATCTAGTAATGGTC
                ************************************************************

DroB797         TAGCGATGAAGCTAACTTTGATGACTATAATGCCAGTGTACTTAGTTTAGTGACATTCCC
DroB869         TAGCGATGAAGCTAACTTTGATGACTATAATGCCAGTGTACTTAGTTTAGTGACATTCCC
                ************************************************************

DroB797         TGACTTGTTCTCCATTATTTTCTATCCCTAACTCTGTTTCGTGCTCTCCTATTCCGTACA
DroB869         TGACTTGTTCTCCATTATTTTCTATCCCTAACTCTGTTTCGTGCTCTCCTATTCCGTACA
                ************************************************************

DroB797         ACTAGTCGGCATGAATCAAAGTCTCAACTAATGGAAGCAAATACAATTTTTTCGAGGAAA
DroB869         ACTAGTCGGCATGAATCAAAGTCTCAACTAATGGAAGCAAATACAATTTTTTCGAGGAAA
                ************************************************************

DroB797         CAAAGCAAATATATTTTTGTTTCTGCATACTTTGATTTTCTCGTGTTTGTCGTTCTTTAT
DroB869         CAAAGCAAATATATTTTTGTTTCTGCATACTTTGATTTTCTCGTGTTTGTCGTTCTTTAT
                ************************************************************

DroB797         GTAAAAAATGTCATGATTACTTTACAGCCCCAACTCTCAGCCTGTCAACAAACATAATGT
DroB869         GTAAAAAATGTCATGATTACTTTACAGCCCCAACTCTCAGCCTGTCAACAAACATAATGT
                ************************************************************

DroB797         CATTTATTCTGGGTAGAATCCTGACCTTGGATGGTTTTGTTCTATTGATCTGAAGTCAAC
DroB869         CATTTATTCTGGGTAGAATCCTGACCTTGGATGGTTTTGTTCTATTGATCTGAAGTCAAC
                ************************************************************

DroB797         TAATCCCGTCTGAAGTTACACTGGTATTATCATAAGTCAGGCTGTCAACTAAGCCTGTTA
DroB869         TAATCCCGTCTGAAGTTACACTGGTATTATCATAAGTCAGGCTGTCAACTAAGCCTGTTA
                ************************************************************

DroB797         ACAAACAGAGCTCATATTGGAGAATGGATGCATTCTTTAACTATTATATATGTGATCAAG
DroB869         ACAAACAGAGCTCATATTGGAGAATGGATGCATTCTTTAACTATTATATATGTGATCAAG
                ************************************************************

DroB797         CGATTGCCGACATATGAAAACGGAAAGAGAACATCAGGATGTGGAGTAAGCATGGGCAAA
DroB869         CGATTGCCGACATATGAAAACGGAAAGAGAACATCAGGATGTGGAGTAAGCATGGGCAAA
                ************************************************************

DroB797         AACATATATCTAACCAGTGATAAGCTGATGAGAACGACTGGGGTTTAAGAATTTTAAACT
DroB869         AACATATATCTAACCAGTGATAAGCTGATGAGAACGACTGGGGTTTAAGAATTTTAAACT
                ************************************************************

DroB797         TAGTGGTCCATGGTCCGGACATCCAATTTAAGCTAGTGTGAACTTAGGACCATACCTTTA
DroB869         TAGTGGTCCATGGTCCGGACATCCAATTTAAGCTAGTGTGAACTTAGGACCATACCTTTA
                ************************************************************

DroB797         TATCTTTTCATGGAACTAGCAACTTAATTTAGTTCTGGTGTGCAGGTTCTAAAGTGCCGG
```

```
DroB869         TATCTTTTCATGGAACTAGCAACTTAATTTAGTTCTGGTGTGCAGGTTCTAAAGTGCCGG
                ************************************************************

DroB797         ACTGTCGCAACGACGAGTTCAGCGATTGGCCCCAATCATTGCTTGCCATTGGGACATTTG
DroB869         ACTGTCGCAACGACGAGTTCAGCGATTGGCCCCAATCATTGCTTGCCATTGGGACATTTG
                ************************************************************

DroB797         GAAATAAGCAGATAGAGGAGGTAGCACAAGTGCAGGATGCTTCCGAGGATGGGCAGTCCA
DroB869         GAAATAAGCAGATAGAGGAGGTAGCACAAGTGCAGGATGCTTCCGAGGATGGGCAGTCCA
                ************************************************************

DroB797         TGCAAGATGCCATCAAGTTTACAGAGGAGGAAGTAGACAAAATACAGAAAGAGTTTGCAA
DroB869         TGCAAGATGCCATCAAGTTTACAGAGGAGGAAGTAGACAAAATACAGAAAGAGTTTGCAG
                *********************************************************** 

DroB797         TGATACTAGCAAGCAAAGACCAAACGGAAACTCATGACCCGCACGATGATGACCAGGTAG
DroB869         TGATACTAGCAAGCAAAGACCAAACGGAAACTCATGACCCGCACGATGATGACCAGGTAG
                ************************************************************

DroB797         CTTCACACAAAAATGTCGATGAGAGCATAAATGAGAAGCACAGGGACCAGCTGTTTAACA
DroB869         CTTCACACAAAAATGTCGATGAGAGCATAAATGAGAAGCACAGGGACCAGCTGTTTAACA
                ************************************************************

DroB797         AGATCGTCATAAGTAAGGCAAAAGATTCACCGGGGAAGAAAGCAAGTACACTCAAGCCGA
DroB869         AGATCGTCATAAGTAAGGCAAAAGATTCACCGGGGAAGAAAGCAAGTACACTCAAGCCGA
                ************************************************************

DroB797         GATCAGTTGCTTCGCTCCTCAAACTACTCATGCCTAAGGGTGGCTTTGCCTCTGCTGTTC
DroB869         GATCAGTTGCTTCGCTCCTCAAACTACTCATGCGTAAGGGTGGCTTTGCCTCTGCTGTTC
                ********************************* **************************

DroB797         CAGATCCAAGGAACTCTTTCCCTCAATCAAGAATGGAAAAGGTACACTTACATTCTTATG
DroB869         CAGATCCAAGGAACTCTTTCCCTCAATCAAGAATGGAAAAGGTACACTTACATTCTTATG
                ************************************************************

DroB797         TACATTTGTAGTTAATTATACCTGTTGACTCTTGAGTAGTATCTTATATATGATTTTCTT
DroB869         TACATTTGTAGTTAATTATACCTGTTGACTCTTGAGTAGTATCTTATATATAATTTTCTT
                *************************************************** ********

DroB797         CCCATTTCAGCTGCTCAAGGCAATACTTCAGAAGAAAATACATCCGCAAAATTCTTCGGC
DroB869         CCCATTTCAGCTGCTCAAGGCAATACTTCAGAAGAAAATACATCCGCAAAATTCTTCGGC
                ************************************************************

DroB797         GCTCGTACCTAGGAGACATTTGGACTGGAAGCCAGATGAGCAAGAGATCAATGAATGCCT
DroB869         GCTCGTACCTAGGAGACATTTGGACTGGAAGCCAGATGAGCAAGAGATCAATGAATGCCT
                ************************************************************

DroB797         TGAGGATGCACTCCGTGATCTAGACGATGATGGCGCAAAATGGGTCAAAACTGATTCAGA
DroB869         TGAGGATGCACTCCGTGATCTAGACGATGATGGCGCAAAATGGGTCAAAACTGATTCAGA
                ************************************************************

DroB797         CTGTAAGTTGCATACTTTGATCCCGAACAAATGACGTTATCATGCACTGAACATCTCTTT
DroB869         CTGTAAGTTGCATACTTTGATCCCGAACAAATGACGTTATCATGCACTGAACATCTCTTT
                ************************************************************

DroB797         ATGCACTGATTCCTTTGAAGAAACTCAGTATGTTATTTTCATTCTTTGCAGTTATTGTGC
DroB869         ATGCACTGATTCCTTTGAAGAAACTCAGTATGTTATTTTCATTCTTTGCAGTTATTGTGC
                ************************************************************

DroB797         TAGAAATGTAAACATGGGTGAATACATTTGTTCAATGAGGCCAGGTGTGCATCTTTTTTC
DroB869         TAGAAATGTAAACATGGGTGAATACATTTGTTCAATGAGGCCAGGTGTGCATCTTTTTTC
                ************************************************************

DroB797         TCAACTCACAGCTATCAGGAGAACTCATGGCTAATATTTATCCTTTATTTTGAGGGTTGT
DroB869         TCAACTCACAGCTATCAGGAGAACTCATGGCTAATATTTATCCTTTATTTTGAGGGTTGT
                ************************************************************

DroB797         GCTTGCAATTTGATTGATTACAATGTGCAGGTTCCAAAGGTCTTGGCAAGCTAGGGATCC
```

```
DroB869          GCTTGCAATTTGATTGATTACAATGTGCAGGTTCCAAAGGTCTTGGCAAGCTAGGGATCC
                 ************************************************************

DroB797          GCGGTGTTATGATATATATATATATAGAGGTGTGAGATATAGTCATCAATGAAGCTATGAAC
DroB869          GCGGTGTTATGATATATATATATATAGAGGTGTGAGATATAGTCATCAATGAAGCTATGAAT
                 ***********************************************************

DroB797          TATGATGTAATGCCCCTCTGTTTCTCACATAAATCATTTGTAATTTGGGTTACTTGGATT
DroB869          TATGATGTAATGCCCCTCTGTTTCTCACATAAATCATTTGTAATTTGGGTTACTTGGATT
                 ************************************************************

DroB797          ATGATAAGAAACTCGCCGGGACAAGCCATTCCTCATGTCTCTGTTTTTCTCTTGTCCTTC
DroB869          ATGATAAGAAACTCGCCGGGACAAGCCATTCCTCATGTCTCTGTTTTTCTCTTGTCCTTC
                 ************************************************************

DroB797          AGACTGTATATTACAAGGTCTGTTACCATTCACATATGAACAGTGATGATGGATATTTCT
DroB869          AGACTGTATATTACAAGGTCTGTTACCATTCACATATGAACAGTGATGATGGATATTTCT
                 ************************************************************

DroB797          GATCCTCCTTGAATGAATTTAATTATGATATAGTTGTTGTTTTATCACATATATTGTCTA
DroB869          GATCCTCCTTGAATGAATTTAATTATGATATAGTTGTTGTTTTATCACATATATTGTCTA
                 ************************************************************

DroB797          CTTAATTTAGATCAGCTTGCAACTTGTGCCTAGTAGATCTGGGTTTCACGCCAGATAGTT
DroB869          CTTAATTTAGATCAGCTTGCAACTTGTGCCTAGTAGATCTGGGTTTCACGCCAGATAGTT
                 ************************************************************

DroB797          AAGAGCCCCTTTGATCTGTAGGATTTGAAGAACCCAGTAACAAAATAAATACAAGATTGT
DroB869          AAGAGCCCCTTTGATCTGTAGGATTTGAAGAACCCAGTAACAAAATAAATACAAGATTGT
                 ************************************************************

DroB797          AAAGAAAAAATGCCCTTTTCAACCATGCACGATTAGAAAACATAAAAATTTGCACTGAAA
DroB869          AAAGAAAAAATGCCCTTTTCAACCATGCACGATTAGAAAACATAAAAATTTGCACTGAAA
                 ************************************************************

DroB797          CATGTTTGAGCTACACGAACGTTCCATGTTGAAACCTGAACTACTGTGCTTGATTTGACG
DroB869          CATGTTTGAGCTACACGAACGTTCCATGTTGAAACCTGAACTACTGTGCTTGATTTGACG
                 ************************************************************

DroB797          ACAACTGAACGTTGAAACCATCTATGTTAAACTTGAACTTCTGAAACCATCTTGAGAAAG
DroB869          ACAACTGAACGTTGAAACCATCTATGTTAAACTTGAACTTCTGAAACCATCTTGAGAAAG
                 ************************************************************

DroB797          CTATGAATATGAAATCAATCCTTTGTCTTTTCGGAGGTGGGG
DroB869          CTATGAATATGAAATCGATCCTTTGTCTTTTCGGAGGTGGGG
                 **************** *************************
```

Figure S1. *TtDro1A* and *TtDro1B* genomic sequence alignment by MUSCLE (3.8). Colours indicate exons in blue; introns in black, UTR regions in red, 5' and 3' regions in green.

Table S1. Primers used in PCR for *TtDro1* subgenomic amplifications and RT-qPCR analysis.

Primer	Sequence (5′→3′)
**TDroA.1F**	CATGAGCCACGCTACAAAGC
**TDroA.1R**	GATTGCTTGACTTCAGCTCATTG
**TDroA.2F**	GATTACTTTATAGCCCCAACCCC
**TDroA.2R**	GAGGTATGGCTTGTCCCAGT
**TDroB.1F**	TGCTCCTCCGAAAAGGGAAT
**TDroB.1R**	GCTTAGTTGACAGCCTGACTTAT
**TDroB.2F**	TCCCGTCTGAAGTTACACTGG
**TDroB.2R**	ATGAGGAATGGCTTGTCCCG
**5UTR.B.F**	CGGGCAAGTAACATACCGATT
**5UTR.B.R**	TGACATTCCCTTTTCGGAGGA
**5UTR.A.1F**	CGATGGGATCCGTCGTGG

**5UTR.A.1R**	GAACCTACGGAGCGTCCTTG
**5UTR.A.2F**	CGCGCCACATCTACACTGG
**5UTR.A.2R**	TTTGATTTGAAGGGGCCGGG
**3UTR.A.1F**	TGTTGAGTGTTGTGCTTGCA
**3UTR.A.1R**	TGTTCTCTAATCGTGGAAGATCA
**3UTR.B.F**	ATGCCCCTCTGTTTCTCACA
**3UTR.B.R**	CCCCACCTCCGAAAAGACAA
**PCRqB.1F**	GAGGTAGCACAAGTGCAGGATG
**PCRqB.1R**	GCGGGTCATGAGTTTCCGT3
**PCRqA.1F**	CAAGCAGAAGCCCATGACT
**PCRqA.1R**	TCTTTTGCCTTACTTATGACGACC
